# SAA/FPR2 Signaling Between Pericentral Hepatocytes and Macrophages Exacerbates Zonated Liver Transplant Injury

**DOI:** 10.1002/advs.202522891

**Published:** 2026-03-29

**Authors:** Feng Zhang, Rong Li, Tingting Wang, Jianhao Zhang, Zhengqi Wu, Qiang You, Xuying Liu, Cuicui Xiao, Jiebin Zhang, Haitian Chen, Jiaqi Xiao, Jia Yao, Jun Zheng, Yingcai Zhang, Hua Li, Shuhong Yi, Yang Yang, Qi Zhang, Xiaofeng Yuan, Yasong Liu

**Affiliations:** ^1^ Department of Hepatic Surgery and Liver Transplantation Center the Third Affiliated Hospital of Sun Yat‐Sen University Guangzhou China; ^2^ Guangdong Provincial Key Laboratory of Liver Disease Research the Third Affiliated Hospital of Sun Yat‐sen University Guangzhou China; ^3^ Cell‐gene Therapy Translational Medicine Research Center the Third Affiliated Hospital of Sun Yat‐Sen University Guangzhou China; ^4^ Department of Anesthesiology the Third Affiliated Hospital of Sun Yat‐Sen University Guangzhou China; ^5^ Department of Hepatobiliary Surgery People's Hospital of Xinjiang Uyghur Autonomous Region Urumqi Xinjiang China; ^6^ Department of General Intensive Care Unit Lingnan Hospital The Third Affiliated Hospital of Sun Yat‐Sen University Guangzhou China

**Keywords:** hepatic ischemia‐reperfusion injury, liver zonation, SAA/FPR2 signaling, liver transplantation, Amilo‐5MER

## Abstract

Liver transplantation (LT) grafts exhibit selective pericentral hepatic ischemia‐reperfusion injury (HIRI), but the zonal mechanism is unclear. To address this, we integrated single‐cell and spatial transcriptomics with chromatin accessibility profiling, proteomics, and bulk RNA‐seq, then validated key findings in clinical LT specimens, mouse HIRI models, and in vitro assays. Across datasets, HIRI disproportionately aggravated pericentral hepatocyte injury and markedly coincided with heightened macrophage crosstalk. Mechanistically, we identified FOXO1‐dominated SAA secretion from pericentral hepatocytes that promotes FPR2^+^ macrophages recruitment and activation, thereby exacerbating pericentral‐predominant hepatocyte injury. Supporting this mechanism, pharmacological inhibition of SAA‐driven proinflammatory activity using the SAA inhibitor Amilo‐5MER reduced macrophage accumulation, alleviated pericentral hepatocyte damage, and mitigated zonal disparities. Collectively, these findings delineate the emerging role of FOXO1/SAA/FPR2 axis in pericentral vulnerability, pinpointing SAA as a tractable therapeutic target to ameliorate zonal injury and improve graft outcomes after LT.

## Graphical abstract

After liver transplantation, ischemia‐reperfusion injury is more severe in pericentral regions. Multiomic analyses of human grafts and mouse models identify FOXO1 activation in pericentral hepatocytes as an upstream driver of SAA secretion. SAA recruits and activates FPR2^+^ macrophages, amplifying local inflammation. Amilo‐5MER inhibits SAA bioactivity, reduces macrophage accumulation, and rebalances zonal injury.

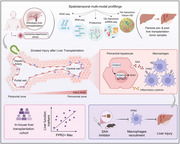

## 1 Introduction

Liver transplantation (LT) is the definitive curative option for end‐stage liver disease and selected hepatic malignancies [1, 2]. Advances in perioperative management, including surgical technique, care protocols, and immunosuppressive regimens, have substantially improved transplant outcomes [3, 4]. However, hepatic ischemia/reperfusion injury (HIRI), characterized by sterile inflammation and hepatocyte apoptosis, persists as an unavoidable pathophysiological event that heightens risks of early graft dysfunction and contributes substantially to post‐transplant morbidity and mortality [5, 6]. HIRI is driven by a multifaceted and highly interconnected network of mechanisms, and despite extensive investigation, definitive and broadly effective therapeutic strategies for LT‐associated HIRI remain limited [5, 7].

The liver exhibits marked spatial heterogeneity at the lobular scale, a feature that is increasingly considered in the design of translational liver models and therapeutic development [8, 9]. This spatial heterogeneity is shaped by metabolic and secretory gradients across the lobule, a framework referred to as liver zonation [10, 11]. These gradients establish three structurally and functionally distinct zones under the paradigm of “liver zonation”: periportal (Zone 1, near the portal veins), midzonal (Zone 2, between the portal and central veins), and pericentral (Zone 3, near the central veins) [12, 13]. Each compartment demonstrates a unique gene expression profile calibrated to zone‐imbalanced microenvironmental cues [14]. This zonal architecture is a key determinant of hepatic pathophysiology, with differential cellular susceptibility producing region‐specific disease patterns across liver disorders. For example, pericentral regions are especially vulnerable to acute acetaminophen or CCl_4_ toxicity, consistent with their dominant role in xenobiotic metabolism [15, 16]. By contrast, periportal regions are more prone to cholestatic injury, in line with stronger activation by damage‐associated molecular patterns [13, 17].

To investigate the zonation‐imbalanced hepatocyte injury patterns in LT‐induced HIRI, we integrated single‐cell RNA sequencing (scRNA‐seq), spatial transcriptomics (ST), ATAC‐seq, proteomics, and bulk RNA‐seq. Our comprehensive analysis revealed that pericentral hepatocytes sustain the most severe in vivo injury post‐LT, despite similar intrinsic susceptibility in vitro. Mechanistically, FOXO1 activity increased in pericentral hepatocytes, directly upregulating *SAA1* transcription and elevating SAA secretion. SAA engaged FPR2 on macrophages, recruited FPR2^+^ macrophages to the pericentral niche, and amplified local inflammatory damage. Therapeutically, Amilo‐5MER, a clinical‐stage inhibitor of SAA pro‐inflammatory activity, reduced FPR2^+^ macrophage accumulation and mitigated HIRI, supporting a tractable strategy for graft protection in LT.

## 2 Results

### 2.1 Multi‐Omics Analysis Identifies Zone‐Imbalanced Hepatocyte Injury Following Human Liver Transplantation

**FIGURE 1:**
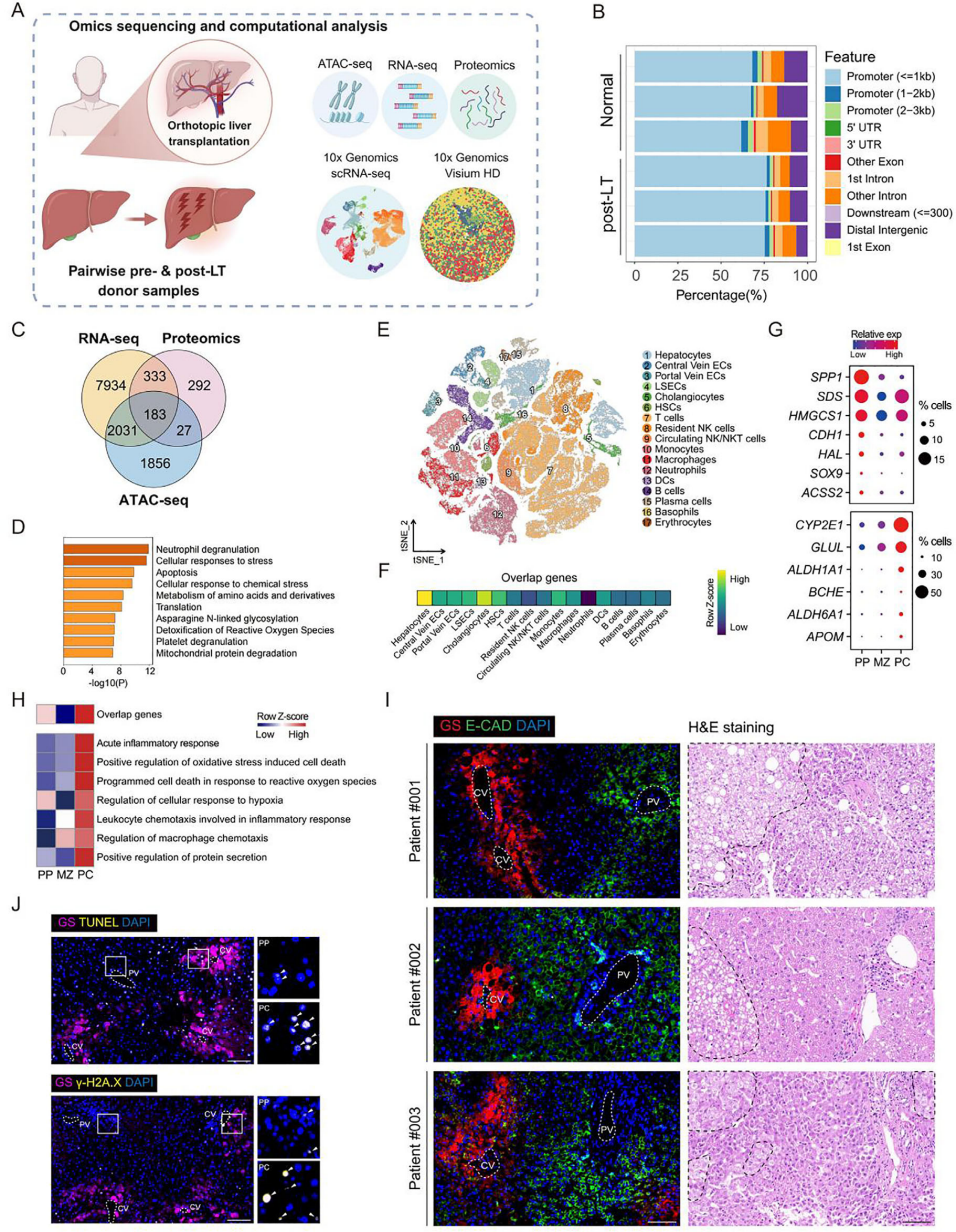
Multi‐omics Analysis Identifies Zone‐imbalanced Hepatocyte Injury Following Human Liver Transplantation. (A) Schematic of the experimental workflow integrating multiple omics to profile pre‐LT and post‐LT donor livers. (B) Stacked bar charts summarizing the genomic annotations of chromatin‐accessible regions. (C) Venn diagram showing the overlap among genes upregulated at the chromatin, transcript, and protein levels in post‐LT livers. (D) Pathway enrichment analysis of the overlap genes defined in panel C. (E) t‐SNE map depicting 17 hepatic cell clusters derived from pre‐ and post‐LT human donor samples. (F) Heatmap displaying the module scores of the panel‐C gene set across hepatic cell types in post‐LT livers. (G) Dot plot of zonation markers used to annotate periportal (PP), midzonal (MZ), and pericentral (PC) hepatocytes. (H) Heatmaps showing module scores of the panel‐C gene set and injury‐related pathways across zonated hepatocyte subsets. (I) Immunofluorescence and H&E staining of post‐LT donor liver on adjacent sections. Glutamine synthetase (GS, red) marks PC hepatocytes; E‐cadherin (E‐CAD, green) marks PP hepatocytes. (J) TUNEL (yellow; upper) and γ‐H2A.X (yellow; lower), co‐stained with GS (magenta). Magnified insets at right display the corresponding TUNEL or γ‐H2A.X signals; triangular arrowheads indicate positive nuclei. CV, central vein; PV, portal vein. Scale bar, 100 µm.

Liver transplantation (LT)‐induced hepatic ischemia/reperfusion injury (HIRI) is a key factor contributing to early graft dysfunction and primary nonfunction. With advancements in sequencing technologies, integrated multi‐omics analysis enables comprehensive exploration of underlying regulatory mechanisms. Here, we analyzed human liver samples collected pre‐ and post‐ LT using ATAC‐seq (Normal = 3, post‐LT = 3), RNA‐seq (Normal = 7, post‐LT = 7), proteomics (Normal = 4, post‐LT = 4), single‐cell RNA sequencing (scRNA‐seq; Normal = 14, post‐LT = 4), and spatial transcriptomics (Normal = 2, post‐LT = 2) to elucidate the primary mechanisms of transplantation‐associated liver injury (Figure 1A).

ATAC‐seq analysis revealed a global increase in chromatin accessibility following LT, particularly around promoter regions, indicative of elevated transcriptional activity (Figure 1B). To further pinpoint key regulatory genes, we integrated ATAC‐seq, RNA‐seq, and proteomic data, identifying 183 genes with coordinated increases in chromatin accessibility, transcript abundance, and protein levels—highlighting their potential central roles in LT‐induced HIRI (Figure 1C). These genes were significantly enriched in inflammation‐related pathways, including stress response and apoptosis (Figure 1D). To identify the cellular sources of these molecular alterations, we performed scRNA‐seq and categorized intrahepatic cells into 17 distinct subpopulations, including hepatocytes, central vein ECs, portal vein ECs, LSECs, cholangiocytes, HSCs, T cells, resident NK cells, circulating NK/NKT cells, monocytes, macrophages, neutrophils, dendritic cells, B cells, plasma cells, basophils, and erythrocytes (Figure 1E and Figure S1A). Following LT, the proportions of monocytes, macrophages, neutrophils, B cells, and plasma cells increased, whereas resident NK cells and circulating NK/NKT cells decreased, suggesting that immune subtypes may play distinct, potentially opposing, roles in transplantation (Figure S1B). Analysis of the 183 genes defined by the intersection of ATAC‐seq, RNA‐seq, and proteomics showed predominant expression in non‐immune cell populations, particularly hepatocytes (Figure 1F). Given this hepatocyte‐centered signal and the well‐recognized regional heterogeneity of hepatocyte injury during liver disease, we next examined whether LT‐associated hepatocyte transcriptional programs differ across lobular zones [10, 13, 16]. Accordingly, hepatocytes were partitioned into periportal, midzonal, and pericentral subsets using canonical zonation markers (Figure 1G) [13, 18]. Notably, the upregulated genes exhibited a strong zonation‐dependent pattern, with the most pronounced increases observed in pericentral hepatocytes. Post‐LT, compared to periportal and midzonal hepatocytes, pericentral hepatocytes displayed significant enrichment in pathways related to inflammation, oxidative stress, hypoxia response, and immune cell chemotaxis (Figure 1H).

These multi‐omics findings were supported by histological validation. Immunofluorescence staining with E‐cadherin (E‐CAD) and glutamine synthetase (GS) confirmed distinct zonal boundaries. Histopathological assessment with hematoxylin and eosin (H&E) staining demonstrated severe structural injury with pericentral predominance (Figure 1I). Consistently, oxidative stress marker γ‐H2A.X and apoptosis marker TUNEL were markedly enriched around the central vein relative to other lobular zones, confirming a pericentral‐predominant pattern of oxidative DNA damage and apoptosis (Figure 1J).

Together, these results identify pericentral hepatocytes as the principal cellular targets of transcriptional reprogramming and injury during human liver transplantation.

### 2.2 Validation of Zone‐Imbalanced Hepatocyte Injury in Mouse Hepatic Ischemia‐Reperfusion Injury (HIRI) Model

**FIGURE 2:**
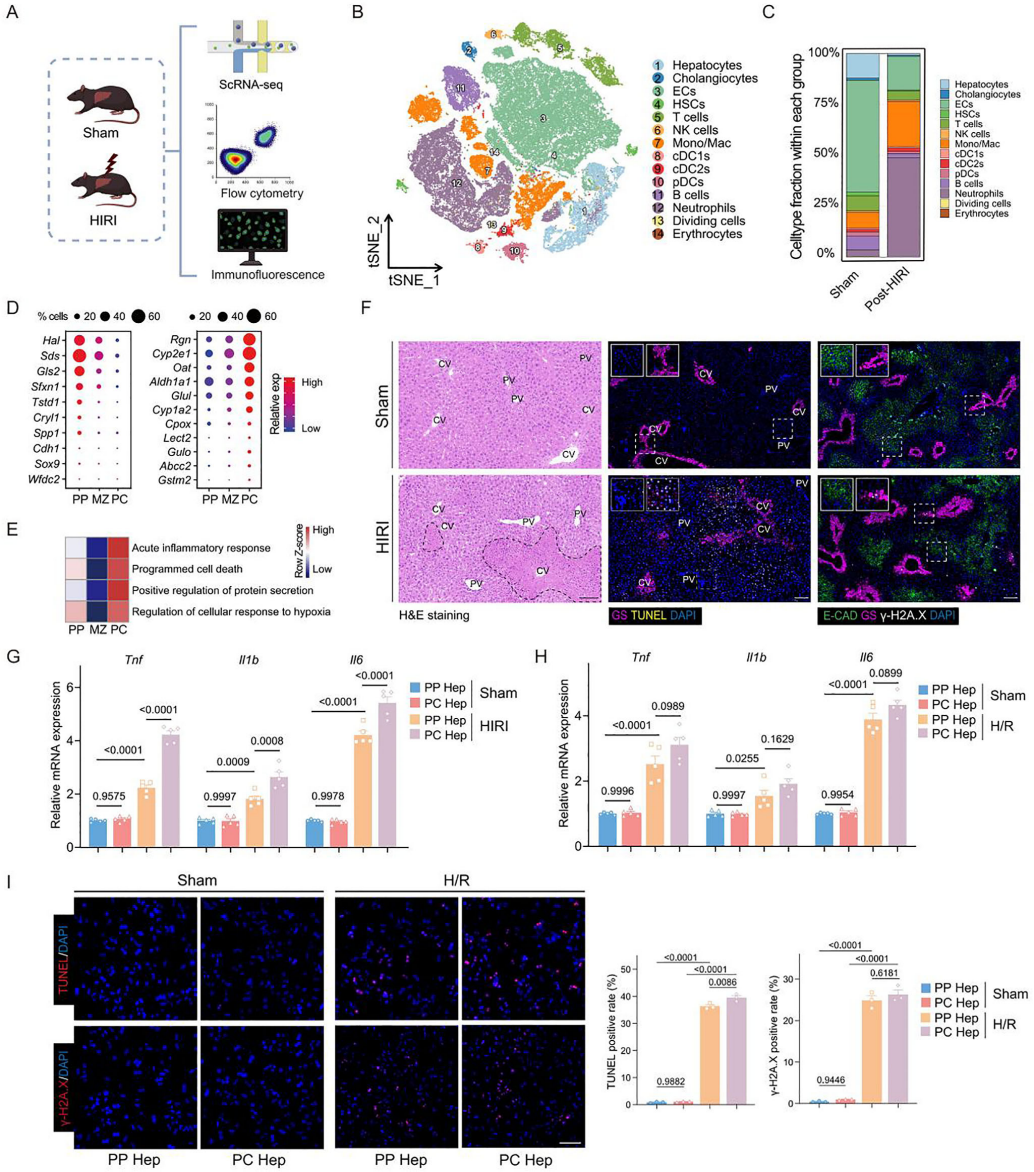
Validation of Zone‐imbalanced Hepatocyte Injury in Mouse Hepatic Ischemia‐Reperfusion Injury (HIRI) Model. (A) Schematic of the experimental workflow using sham‐operated and post‐HIRI mouse livers. (B) t‐SNE map depicting 14 hepatic cell clusters derived from sham and post‐HIRI mouse livers. (C) Stacked bar charts showing cell‐type composition changes. (D) Dot plot of zonation markers used to annotate PP, MZ, and PC hepatocytes. (E) Heatmaps showing module scores of injury‐related pathways across zonated hepatocyte subsets. (F) H&E (left), TUNEL (middle), and γ‐H2A.X (right) staining, co‐stained with GS and E‐CAD in mouse livers. (G,H) RT‐qPCR showing expression changes of *Tnf*, *Il1b*, and *Il6* in PP compared to PC hepatocytes from in vivo HIRI vs. sham models (G) and upon in vitro hypoxia‐reoxygenation (H/R) intervention (H). *n* = 5, per group. (I) Primary PP and PC hepatocytes subjected to H/R intervention. Representative images (left) and quantification (right) of TUNEL and γ‐H2A.X staining are shown (*n* = 3, per group). Scale bar, 100 µm. *p* < 0.05 was considered significant.

To corroborate the human findings, we established a mouse HIRI model (Sham = 3; HIRI = 3) and integrated scRNA‐seq with spatial and histological assays (Figure 2A). ScRNA‐seq identified 14 distinct hepatic cell populations, with major populations conserved between mice and humans (Figure 2B and Figure S1C). Consistent with findings in human LT, proportions of Monocytes/Macrophages (Mono/Mac) and neutrophils were markedly increased in mouse livers post‐HIRI (Figure 2C and Figure S1D). Hepatocytes were stratified into periportal, midzonal, and pericentral groups based on zone‐specific markers (Figure 2D). Pathway analysis showed that inflammatory, hypoxia response, and cell death programs had the highest activity in pericentral hepatocytes after HIRI (Figure 2E). Spatial immunostaining with E‐CAD and GS accurately delineated periportal–pericentral boundaries in mouse livers, and histology showed a pericentral‐predominant pattern of structural injury, apoptosis, and oxidative DNA damage (Figure 2F and Figure S2A).

To further validate zonal specificity at the cellular level, we prospectively purified hepatocytes by fluorescence‐activated cell sorting (FACS) into periportal and pericentral fractions and confirmed purity by qPCR, which demonstrated *Cyp2e1* enrichment in pericentral hepatocytes and *Sds* enrichment in periportal hepatocytes (Figure S2B). In vivo, pericentral hepatocytes mounted a greater induction of pro‐inflammatory cytokines (*Tnf*, *Il1b*, *Il6*) than periportal hepatocytes after HIRI (Figure 2G). By contrast, when periportal and pericentral hepatocytes isolated from uninjured livers were subjected to hypoxia/reoxygenation (H/R) in vitro, cytokine induction and TUNEL/γ‐H2A.X staining levels were comparable between groups (Figure 2H,I).

In the mouse HIRI model, transcriptomic profiling coupled with zonal mapping validates a pericentral‐predominant pattern of hepatocyte injury, consistent with our observations in human LT. The inability of in vitro H/R to reproduce the in vivo zonal disparity suggests that this vulnerability cannot be explained solely by hepatocyte‐intrinsic susceptibility, implicating an additional contribution from extrinsic microenvironmental cues.

### 2.3 Macrophages Exacerbate Pericentral‐Predominant Hepatocyte Injury After Liver Transplantation and Correlate With FPR2^+^ Macrophage Accumulation

**FIGURE 3:**
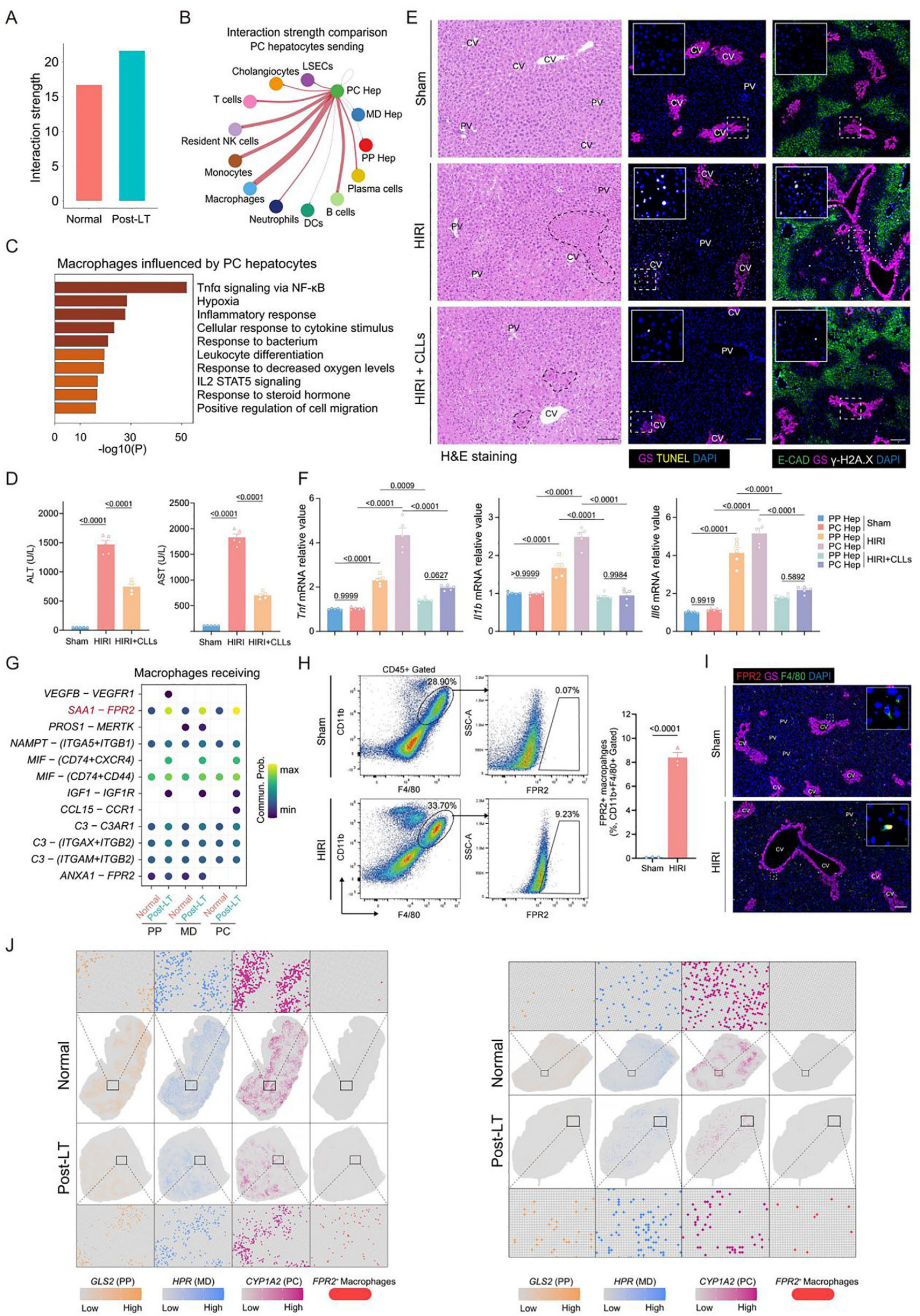
Macrophages exacerbate pericentral‐predominant hepatocyte injury after liver transplantation and correlate with FPR2^+^ macrophage accumulation. (A) Interaction strength within the hepatic microenvironment in normal and post‐LT human donor livers. (B) Circle plot illustrating differential intercellular signaling interactions sent by PC hepatocytes in humans. Line color indicates signaling interaction change in post‐LT donor livers (red, upregulation; blue, downregulation). Line thickness represents changes in interaction strength. (C) Enrichment analysis of differentially expressed genes in macrophages inferred to be modulated by PC hepatocyte signaling during LT. (D) Serum ALT and AST levels across groups (*n* = 5, per group). (E) H&E (left), TUNEL (middle), and γ‐H2A.X (right) staining, co‐stained with GS and E‐CAD in mouse livers. (F) RT‐qPCR showing expression changes of *Tnf*, *Il1b*, and *Il6* in PP and PC hepatocytes across groups (*n* = 5, per group). (G) Bubble plot displaying differentially regulated ligand–receptor pairs mediating zonated hepatocyte‐to‐macrophage signaling in post‐LT vs. normal human donor livers. (H) Representative flow‐cytometry plots (left) and quantification (right) of FPR2^high^ macrophages in livers from sham and HIRI mice (*n* = 3, per group). (I) IF staining showing accumulation of FPR2^+^ macrophages around PC hepatocytes. (J) Spatial transcriptomic maps showing the expression of *GLS2* (periportal), *HPR* (midzonal), and *CYP1A2* (pericentral) in normal and post‐LT donor livers, together with the spatial distribution of FPR2^+^ macrophages. Representative regions were magnified to highlight areas enriched for pericentral markers. Scale bar, 100 µm. *p* < 0.05 was considered significant.

Motivated by the dissociation between in vivo zonal disparity and in vitro hepatocyte responses, we next assessed cell–cell communication to identify extrinsic contributors to pericentral vulnerability. Overall cell–cell interaction strength across the hepatic microenvironment was markedly increased after injury in both human post‐LT livers and mouse post‐HIRI livers (Figure 3A and Figure S3A). Across these datasets, directional signaling analysis further indicated that pericentral hepatocytes exhibited the strongest outgoing interactions toward macrophages (Figure 3B and Figure S3B). Correspondingly, gene enrichment analyses of macrophage transcriptional programs inferred to be downstream of pericentral hepatocyte signaling revealed inflammatory signatures, consistent with macrophage responses contributing to the amplification of zone‐imbalanced injury (Figure 3C).

To validate this hypothesis, macrophages were depleted in mice using clodronate liposomes (CLLs) prior to HIRI (Figure S3C). Depletion reduced serum ALT and AST and mitigated histological injury on H&E, TUNEL, and γ‐H2A.X staining, eliminating the pericentral–periportal disparity in injury severity (Figure 3D,E). qPCR of FACS‐purified periportal and pericentral hepatocytes showed comparable expression of injury markers after depletion, further indicating that macrophage depletion markedly blunts the zonal disparity in injury severity (Figure 3F).

To define the recruitment signal, ligand–receptor analysis revealed marked upregulation of *SAA1*–*FPR2* signaling after LT, with the strongest interactions observed from pericentral hepatocytes to macrophages (Figure 3G). Concordantly, *FPR2* expression in macrophages increased post‐LT (Figure S3D). Flow cytometry and immunofluorescence detected essentially no FPR2^+^ macrophages in normal mouse liver and a robust accumulation after HIRI (Figure 3H,I and Figure S4A). Spatial transcriptomics of human liver further demonstrated that FPR2^+^ macrophages preferentially clustered within pericentral regions after LT, rather than in periportal or midzonal regions (Figure 3J and Figure S4B) [18].

Collectively, these data demonstrate that macrophage responses are required for the full manifestation of pericentral‐predominant injury after LT, and identify *SAA1–FPR2* as a prominent hepatocyte‐to‐macrophage signaling axis associated with macrophage accumulation and inflammatory activation.

### 2.4 Pericentral Hepatocyte‐Derived SAA Drives FPR2^+^ Macrophage Recruitment to Exacerbate HIRI

Proteomic profiling of human livers before and after LT identified SAA1 among the 10 most upregulated proteins post‐LT, implicating SAA1 in LT‐induced HIRI (Figure S5A). ScRNA‐seq localized *SAA1* predominantly to hepatocytes, with the highest abundance in pericentral hepatocytes (Figure S5B,C). This pericentral enrichment was conserved in mice, where *Saa1* levels were markedly increased in pericentral hepatocytes post‐HIRI (Figure S5D). These findings indicate that pericentral hepatocytes possess a heightened capacity for SAA production and secretion relative to other lobular zones.

**FIGURE 4:**
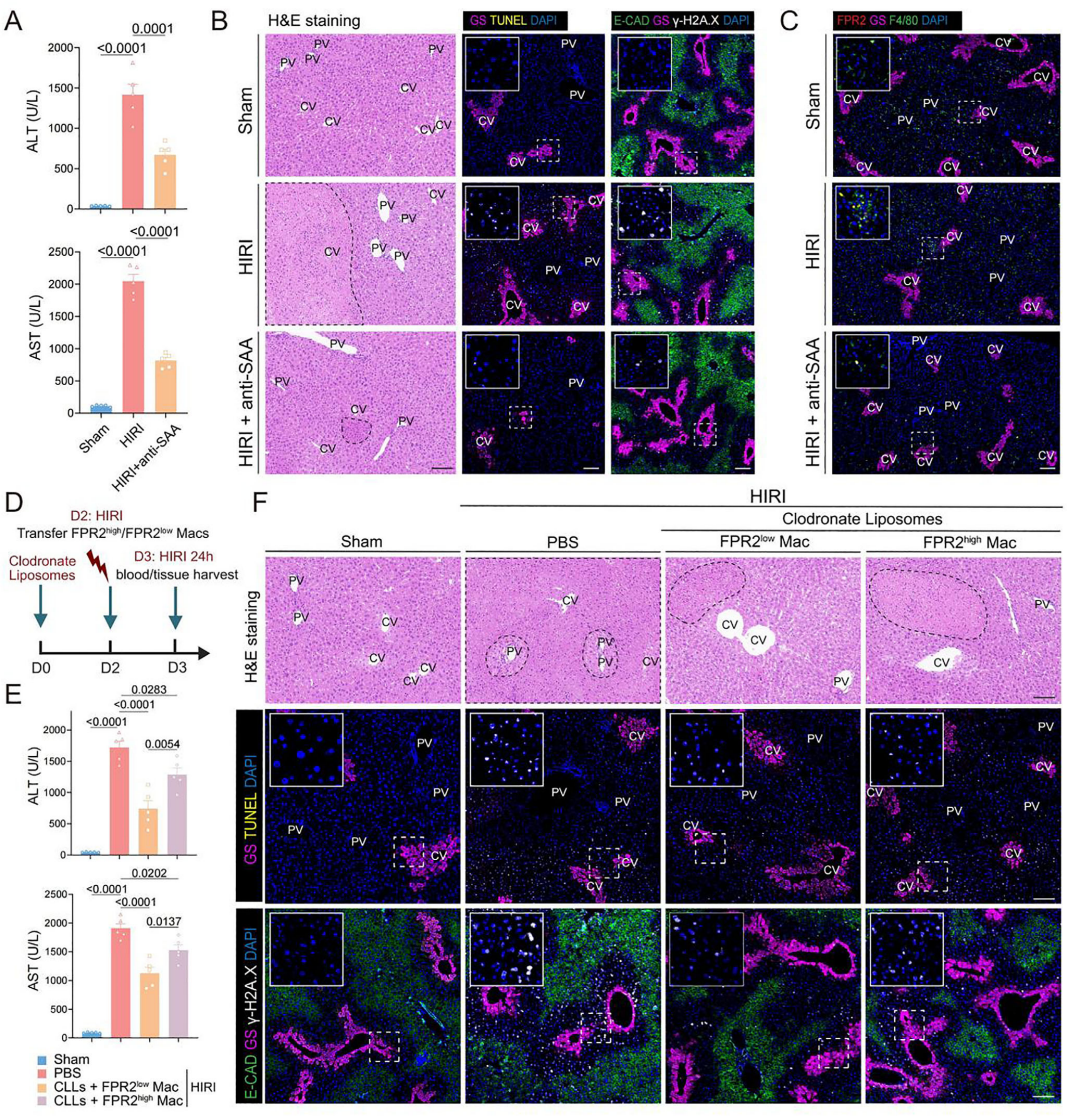
Pericentral Hepatocyte‐Derived SAA Drives FPR2^+^ Macrophage Recruitment to Exacerbate HIRI. (A) Serum ALT and AST levels across groups (*n* = 5, per group). (B) H&E (left), TUNEL (middle), and γ‐H2A.X (right) staining, co‐stained with GS and E‐CAD in mouse livers. (C) Immunofluorescence showing accumulation of FPR2^+^ macrophages around PC hepatocytes; magnified views are shown at the upper left of each panel. (D) Schematic displaying the experimental design. Mice were pretreated with CLLs to deplete macrophages for 48 h, and the HIRI model was subsequently established, followed by a single intravenous injection of FPR2^high^ or FPR2^low^ macrophages. (E) Serum ALT and AST levels across groups (*n* = 5, per group). (F) H&E (upper), TUNEL (middle), and γ‐H2A.X (lower) staining, co‐stained with GS and E‐CAD in mouse livers. Scale bar, 100 µm. *p* < 0.05 was considered significant.

To determine whether increased SAA secretion from pericentral hepatocytes causally drives FPR2^+^ macrophage recruitment and pericentral hepatocyte injury, we administered SAA‐neutralizing antibodies in the mouse HIRI model. SAA neutralization significantly improved liver function, with lower serum ALT and AST (Figure 4A). Histological analyses by H&E, TUNEL, and γ‐H2A.X staining showed attenuated hepatocyte injury and eliminated the zonal disparity in injury severity (Figure 4B). Consistently, blockade of SAA markedly reduced the accumulation of FPR2^+^ macrophages around pericentral hepatocytes (Figure 4C). In addition, to minimize potential confounding from circulating SAA and to directly test the contribution of hepatocyte‐derived SAA, we performed hepatocyte‐specific SAA knockdown using AAV8‐TBG‐*Saa1‐shRNA*. Efficient SAA silencing was confirmed in primary hepatocytes isolated from AAV8‐TBG‐*Saa1‐shRNA* vs. control mice after HIRI (Figure S5E). Following HIRI, hepatocyte‐specific SAA knockdown lowered serum ALT and AST and attenuated histological injury on H&E, TUNEL, and γ‐H2A.X staining, while eliminating zonal disparities (Figure S5F,G). In line with suppression of the SAA–FPR2 axis, the accumulation of FPR2+ macrophages declined and no longer showed pericentral enrichment (Figure S5H).

Subsequently, we explored whether FPR2^high^ macrophages possess an activated inflammatory phenotype capable of exacerbating liver injury. After macrophage depletion in mice, adoptive transfer experiments with FPR2^high^ and FPR2^low^ macrophages were conducted in mice undergoing HIRI (Figure 4D). Both macrophage subsets accumulated substantially in the liver post‐transfer (Figure S6A). Notably, adoptive transfer of FPR2^high^ macrophages significantly increased serum ALT and AST relative to transfer of FPR2^low^ macrophages, indicating exacerbated liver injury (Figure 4E). Histology (H&E, TUNEL, and γ‐H2A.X) showed that transfer of FPR2^low^ macrophages abrogated the zonal disparity, whereas transfer of FPR2^high^ macrophages aggravated injury with a clear pericentral predominance (Figure 4F). To further strengthen the causal link between macrophage FPR2 expression and zonated liver injury, we next performed macrophage‐specific FPR2 knockdown using AAV8‐F4/80‐*Fpr2‐*shRNA. Efficient FPR2 silencing was confirmed in primary macrophages isolated from AAV8‐F4/80‐*Fpr2‐*shRNA vs. control mice after HIRI (Figure S6B,C). Following HIRI, FPR2 knockdown reduced serum ALT and AST and alleviated histological injury on H&E, TUNEL, and γ‐H2A.X staining, while eliminating zonal disparities (Figure S6D,E).

Collectively, these findings establish SAA as a key mediator that concentrates FPR2^+^ macrophages in the pericentral region, thereby exacerbating pericentral hepatocytes injury post‐HIRI.

### 2.5 FOXO1 Activation Directly Induces SAA1 Transcription and SAA Secretion during HIRI

**FIGURE 5:**
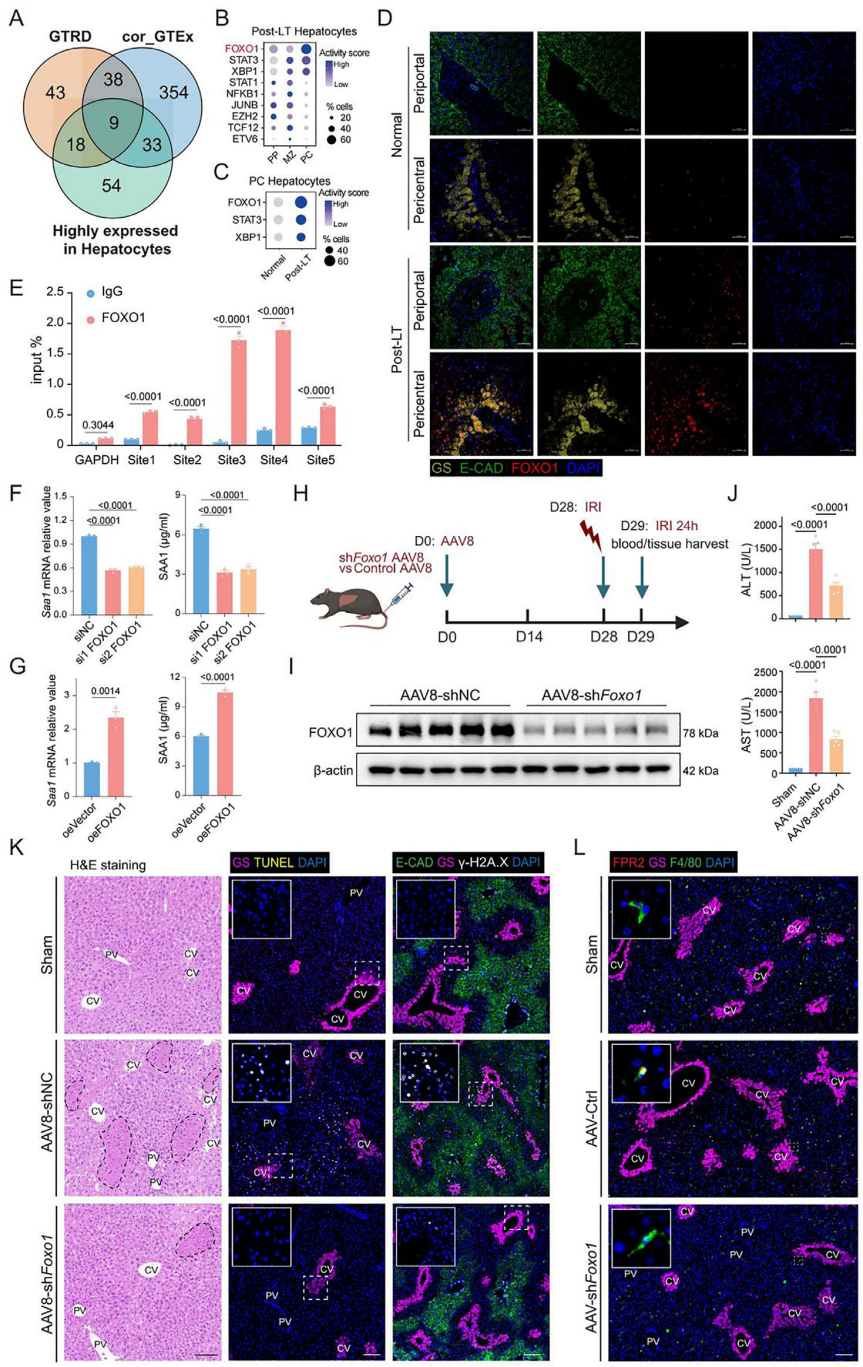
FOXO1 Activation Directly Induces SAA1 Transcription and SAA Secretion During HIRI. (A) Venn diagram showing the intersection of GTRD and cor_GTEx to nominate upstream transcription factors of *SAA1*, filtered for high hepatocyte expression. (B) Dot plot of inferred transcriptional regulatory activity of candidate transcription factors across zonated hepatocytes. (C) Dot plot of inferred regulon activity in PC hepatocytes from normal vs. post‐LT livers. (D) Confocal images of pre‐ and post‐LT donor livers showing co‐staining for GS, E‐CAD, and FOXO1. Scale bar, 50 µm. (E) ChIP‐qPCR demonstrating FOXO1 occupancy at the *Saa1* promoter in mouse primary hepatocytes (*n* = 3, per group). (F,G) *Saa1* mRNA expression level by RT‐qPCR (left) and secreted SAA1 protein level in culture supernatants by ELISA (right), following FOXO1 knockdown (F) or overexpression (G) in mouse primary hepatocytes (*n* = 3, per group). (H) Schematic of the experimental design: AAV8‐TBG‐*Foxo1*‐shRNA or control AAV8 delivered via tail vein 4 weeks before HIRI induction. (I) Immunoblot analysis of primary hepatocytes from AAV8‐TBG‐*Foxo1*‐shRNA vs. control mice (*n* = 5, per group). (J) Serum ALT and AST levels (*n* = 5, per group). (K) H&E (left), TUNEL (middle), and γ‐H2A.X (right) staining, co‐stained with GS and E‐CAD in mouse livers. Scale bar, 100 µm. (L) IF staining showing the distribution of FPR2^+^ macrophages around PC hepatocytes. Scale bar, 100 µm. *p* < 0.05 was considered significant.

To determine the mechanism underlying increased SAA production in pericentral hepatocytes, we prioritized upstream transcription factors predicted to regulate *SAA1*. Binding predictions from GTRD were intersected with GTEx‐based coexpression (cor_GTEx) and then filtered for factors highly expressed in hepatocytes, yielding nine candidates (Figure 5A). Among these, FOXO1, STAT3, and XBP1 showed the strongest inferred regulatory activity in pericentral hepatocytes, with FOXO1 ranking highest (Figure 5B). Concordantly, bulk RNA‐seq showed significant upregulation of FOXO1 post‐LT, whereas STAT3 and XBP1 did not change significantly (Figure S7A). Inferred transcriptional activity likewise increased selectively in pericentral hepatocytes post‐LT (Figure 5C). Consistent with these data, confocal microscopy showed elevated FOXO1 in graft hepatocytes post‐LT, with predominant nuclear localization that was most pronounced in pericentral hepatocytes (Figure 5D and Figure S7B).

Extending these observations to mice, scRNA‐seq in the HIRI model confirmed post‐HIRI activation of FOXO1 in pericentral hepatocytes (Figure S7C). Primary hepatocytes isolated from normal and HIRI livers revealed increased FOXO1 at both mRNA and protein levels after reperfusion (Figure S7D). Likewise, primary hepatocytes subjected to H/R stimulation in vitro also exhibited marked increases in FOXO1 protein and mRNA levels, indicating that H/R directly induces FOXO1 upregulation in hepatocytes (Figure S7E). To determine whether this increase corresponded to enhanced transcriptional activity, nuclear–cytoplasmic fractionation assays and immunofluorescence staining were performed, both confirming increased nuclear localization of FOXO1 following H/R (Figure S7F). To investigate potential transcriptional regulation, we performed bioinformatic screening using the JASPAR database, which predicted five FOXO1 binding sites within the *SAA1* promoter region (Figure S8A). Subsequently, chromatin immunoprecipitation followed by quantitative PCR (ChIP‐qPCR) was performed and confirmed FOXO1 binding at all five predicted sites, with the most robust enrichment observed at sites 3 and 4 (Figure 5E). These results indicate that FOXO1 directly binds the *SAA1* promoter, thus demonstrating its role as a direct transcriptional regulator of *SAA1*. To functionally confirm this regulatory axis, we first manipulated FOXO1 in cultured hepatocytes. FOXO1 knockdown reduced SAA1 expression and lowered secreted SAA, whereas FOXO1 overexpression increased both readouts (Figure S8B,C and Figure 5F,G). Extending these observations in vivo, hepatocyte‐specific FOXO1 knockdown via AAV8‐TBG‐*Foxo1‐*shRNA led to efficient FOXO1 silencing in the liver four weeks after administration (Figure 5H–I and Figure S8D). Following HIRI, FOXO1 knockdown markedly reduced hepatocellular SAA expression and, in parallel, decreased serum ALT and AST and alleviated histological injury on H&E, TUNEL, and γ‐H2A.X staining, thereby eliminating zonal disparities (Figure 5J,K and Figure S8E). Consistent with suppression of the SAA–FPR2 axis, the accumulation of FPR2^+^ macrophages declined and lost its pericentral enrichment (Figure 5L).

Collectively, the data establish that increased FOXO1 transcriptional activity in pericentral hepatocytes during HIRI upregulates SAA production and release, leading to FPR2^+^ macrophage recruitment and consequent pericentral‐predominant hepatocyte injury.

### 2.6 Clinical Relevance of Hepatocyte FOXO1 and FPR2^+^ Macrophages Contribute to Post‐Transplantation Liver Injury

**FIGURE 6:**
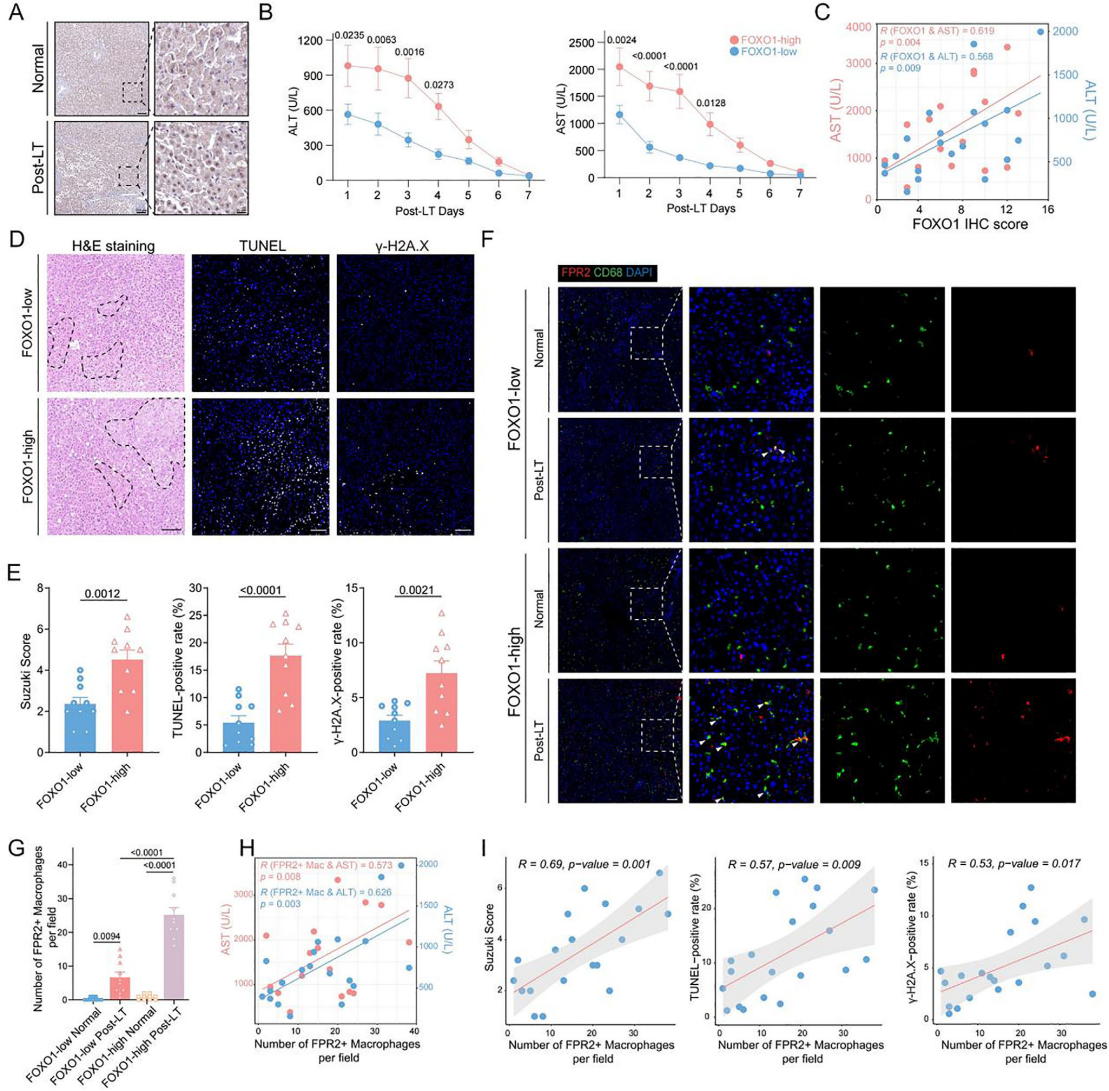
Clinical Relevance of Hepatocyte FOXO1 and FPR2^+^ Macrophages Contribute to Post‐Transplantation Liver Injury. (A) Representative images for immunohistochemical (IHC) staining of FOXO1 in normal and post‐LT human donor livers. (B) The serum ALT and AST levels of liver transplant recipients with high vs. low FOXO1 expression level (*n* = 10, per group) during postoperative day 1–7 (POD1‐7). (C) Correlations between FOXO1 IHC scores and respective serum ALT/AST levels in POD1 (*n* = 20). (D) H&E (left), TUNEL (middle), and γ‐H2A.X (right) staining in post‐LT donor livers, stratified by FOXO1‐high vs. FOXO1‐low LT recipients. (E) Quantification of Suzuki injury scores (left), TUNEL‐positive rates (middle), and γ‐H2A.X‐positive rate (right) in FOXO1‐high vs. FOXO1‐low post‐LT donor livers (*n* = 10, per group). (F) IF staining showing the distribution of FPR2^+^ macrophages in FOXO1‐high vs. FOXO1‐low post‐LT donor livers. (G) Quantification of FPR2^+^ macrophages per field across FOXO1‐high and FOXO1‐low donor livers (*n* = 10, per group). (H) Correlations between FPR2^+^ macrophage counts and serum ALT/AST levels in POD1 (*n* = 20). (I) Correlations between FPR2^+^ macrophage counts and post‐LT histological injury metrics (*n* = 20): Suzuki score (left), TUNEL‐positive rate (middle), and γ‐H2A.X‐positive rate (right). Scale bar, 100 µm. *p* < 0.05 was considered significant.

To evaluate the clinical relevance of this mechanism, we first assessed FOXO1 expression in normal and post‐LT human donor livers using immunohistochemistry (IHC). The proportion of FOXO1^+^ hepatocytes was markedly increased post‐LT (Figure 6A). Post‐LT specimens were then stratified into FOXO1‐high and FOXO1‐low groups according to the FOXO1 IHC score. The FOXO1‐low group exhibited better early postoperative liver function than the FOXO1‐high group, reflected by lower serum ALT and AST at multiple time points (Figure 6B). Consistently, ALT and AST levels on postoperative day 1 correlated positively with the FOXO1 IHC score (Figure 6C). In parallel, histological injury and hepatocellular death were significantly greater in the FOXO1‐high group (Figure 6D,E). As a downstream target of FOXO1, we next examined SAA. Immunofluorescence co‐staining demonstrated that both FOXO1 and SAA were markedly increased in post‐LT liver tissue and showed prominent spatial colocalization (Figure S9A). Moreover, by analyzing paired serum samples collected from donors before transplantation and recipients after transplantation, we found that serum SAA1 levels were significantly elevated post‐LT and scaled with the magnitude of postoperative hepatocellular injury, as reflected by ALT and AST (Figure S9B,C).

We next evaluated FPR2^+^ macrophages. In normal donor livers, FPR2^+^ macrophages were relatively infrequent; their proportions increased after LT in both groups, with a more pronounced rise in the FOXO1‐high group (Figure 6F,G). Across post‐LT samples, the proportion of FPR2^+^ macrophages correlated positively with liver dysfunction, as indicated by higher serum ALT and AST, and likewise with histological injury and hepatocyte death (Figure 6H,I).

Collectively, these data establish that elevated hepatocyte FOXO1 expression, increased SAA abundance and release, and enhanced FPR2^+^ macrophage infiltration are associated with more severe post‐transplant graft injury, supporting the clinical relevance of the FOXO1/SAA/FPR2 axis.

### 2.7 Amilo‐5MER Reduces Pericentral Hepatocyte Injury by Targeting SAA in Hepatic Ischemia‐Reperfusion Injury

**FIGURE 7:**
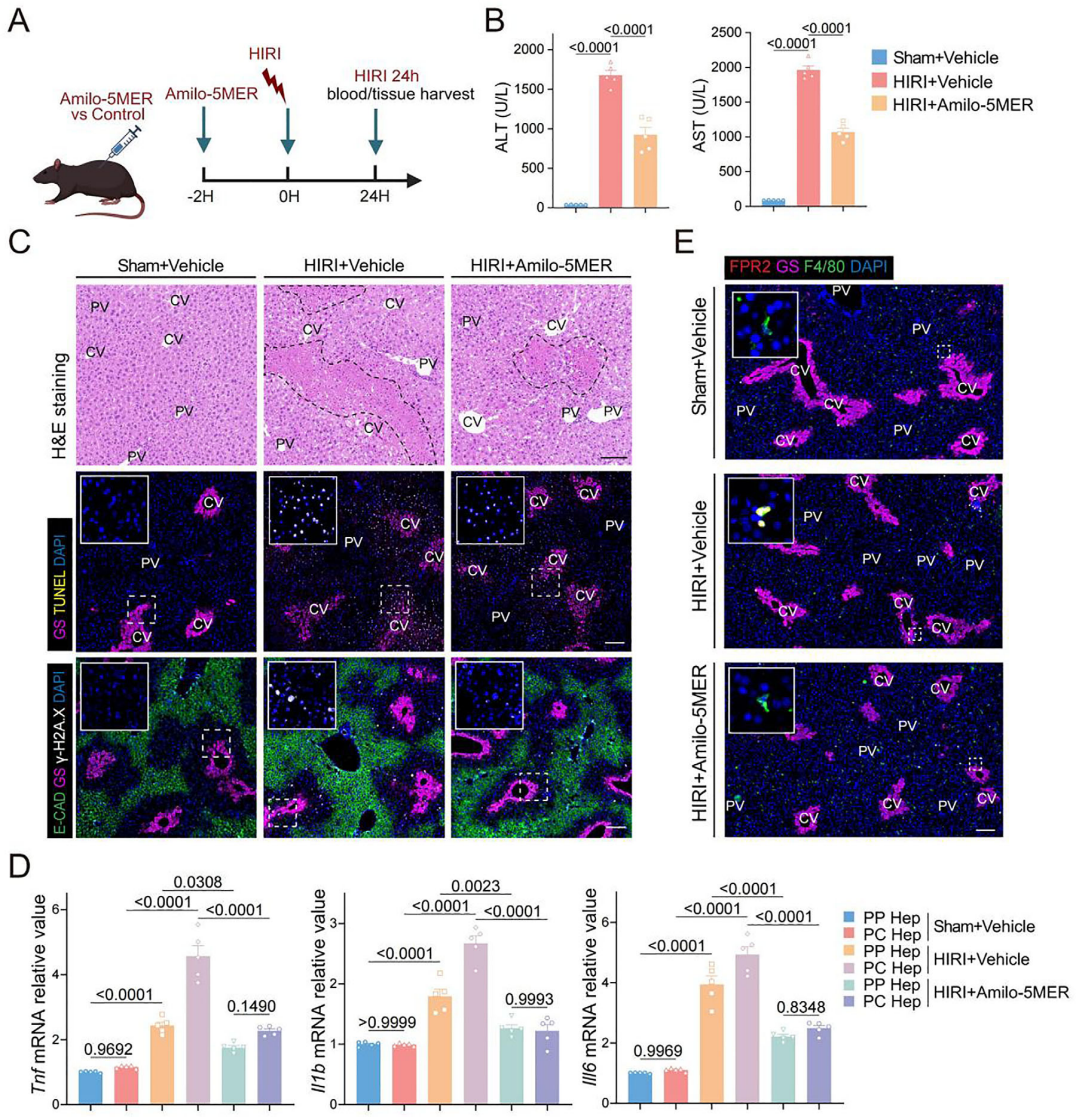
Amilo‐5MER Reduces Pericentral Hepatocytes Injury by Targeting SAA in Hepatic Ischemia‐Reperfusion Injury. (A) Schematic displaying the experimental design. Mice were pretreated with Amilo‐5MER for 2 h, and the HIRI model was subsequently established. (B) The serum ALT and AST levels in each group (*n* = 5, per group). (C) H&E (upper), TUNEL (middle), and γ‐H2A.X (lower) staining, co‐stained with GS and E‐CAD in mouse livers. (D) RT‐qPCR showing expression changes of *Tnf*, *Il1b*, and *Il6* in PP and PC hepatocytes across groups (*n* = 5, per group). (E) IF staining showing the distribution of FPR2^+^ macrophages around PC hepatocytes in mouse livers. Scale bar, 100 µm. *p* < 0.05 was considered significant.

Amilo‐5MER, a peptide that inhibits the pro‐inflammatory activity of SAA and has completed a Phase I safety assessment (NCT05857215), is at a more advanced stage of clinical development than unvalidated anti‐SAA antibodies [19]. To further functionally support its capacity to suppress the SAA–FPR2 axis, we sorted FPR2^high^ and FPR2^low^ macrophages by flow cytometry and performed transwell migration assays. Amilo‐5MER markedly attenuated the chemotactic recruitment of FPR2^high^ macrophages toward H/R‐stimulated hepatocytes, whereas its effect on FPR2^low^ macrophages was minimal, consistent with selective inhibition of SAA‐FPR2‐dependent chemotaxis (Figure S10A,B). Accordingly, we evaluated its efficacy in a murine HIRI model by administering Amilo‐5MER intraperitoneally and comparing outcomes with vehicle‐treated controls (Figure 7A). Serum ALT and AST were lower in the Amilo‐5MER group, indicating improved liver function (Figure 7B). Histopathological assessment by H&E, TUNEL, and γ‐H2A.X staining showed attenuated hepatocellular injury and eliminated the pericentral predominance observed in controls (Figure 7C). At the transcriptional level, qPCR of periportal and pericentral hepatocytes showed lower expression of pro‐inflammatory mediators (*Tnf*, *Il1b*, *Il6*) in the Amilo‐5MER‐treated group with no residual zonal differences (Figure 7D). Consistent with inhibition of the SAA‐FPR2 axis, Amilo‐5MER significantly attenuated the pericentral enrichment of FPR2^+^ macrophages in HIRI (Figure 7E).

In summary, pharmacologic targeting of SAA with Amilo‐5MER ameliorates HIRI, equalizes zonal susceptibility, and limits FPR2^+^ macrophage accumulation, thereby protecting pericentral hepatocytes and supporting further clinical evaluation.

## 3 Discussion

A limited understanding of parenchymal and non‐parenchymal cell dynamics within liver grafts has hindered the development of effective therapies for LT‐induced HIRI [20, 21]. By integrating scRNA‐seq, spatial transcriptomics, ATAC‐seq, proteomics, and bulk RNA‐seq, we constructed a spatiotemporal atlas of graft remodeling and identified pericentral hepatocytes as the predominant injury compartment. These cells upregulated SAA and delivered dominant paracrine signals to macrophages, recruiting and activating FPR2^+^ macrophages to establish a pericentral pro‐inflammatory niche. Upstream, FOXO1 activity was increased in pericentral hepatocytes and drove SAA overexpression, initiating this pathogenic intercellular cascade. Therapeutically, Amilo‐5MER blockade of SAA mitigated liver injury, reduced FPR2^+^ macrophage accumulation, and attenuated zonal vulnerability, providing proof of concept for clinical translation.

Zonal pathology is a pivotal research focus in hepatology, reflecting how metabolic heterogeneity along the portal‐to‐central axis generates zone‐specific vulnerability and distinct cellular programs [10, 18, 22, 23]. Across liver diseases, pericentral hepatocytes exhibit preferential sensitivity to drug‐induced toxicity and alcohol‐associated liver disease, periportal immune programs, including CD74^+^ macrophages, have been linked to metabolic dysfunction‐associated steatohepatitis, and midzonal hepatocytes have been implicated in homeostatic and regenerative functions [10, 12, 24, 25, 26]. In LT‐induced HIRI, preferential pericentral injury has been reported, yet the mechanistic basis for this spatial bias remains incompletely defined. Although canonical physiological determinants, including perfusion and oxygenation gradients and metabolic zonation, are often cited to contextualize lobular vulnerability, their relationship to immune microenvironmental programs that could amplify injury has not been systematically delineated. To address this underexplored aspect, our data support a spatially localized, macrophage‐driven amplification process that preferentially exacerbates pericentral injury. Integrative cell–cell communication analyses across human post‐transplant and mouse HIRI datasets consistently prioritized pericentral hepatocyte‐to‐macrophage signaling as a dominant interaction axis. Functionally, macrophage depletion markedly reduced the pericentral‐to‐periportal disparity despite persistence of underlying lobular gradients. We further nominate pericentrally enriched FPR2^+^ macrophages as a key effector subset, supported by adoptive transfer experiments. Together, these results indicate that zonal injury in LT‐induced HIRI reflects not only pre‐existing physiological gradients but also an actively configured immune microenvironment, and they position pericentral hepatocyte–macrophage crosstalk as a tractable axis for mechanistic and therapeutic interrogation.

Mechanistically, our findings support a FOXO1–SAA–FPR2 signaling axis that connects pericentral hepatocyte stress to macrophage recruitment and inflammatory amplification. In this framework, stressed pericentral hepatocytes increase SAA secretion, which preferentially recruits and enrich FPR2^+^ macrophages, consistent with evidence that SAA engages FPR2 and can drive pro‐inflammatory activation in immune cell [27, 28, 29, 30]. Although FPR2 signaling is ligand‐dependent and can elicit divergent programs, the robust induction of SAA in HIRI is consistent with SAA contributing substantially to FPR2 engagement and promoting chemotactic and pro‐inflammatory signaling during the early injury phase. Upstream, increased FOXO1 nuclear localization and transcriptional activity in pericentral hepatocytes provide a mechanistic basis for SAA1 upregulation, consistent with direct binding of FOXO1 to the SAA1 promoter. Notably, FOXO1 has been reported to have divergent roles across hepatic cell types in HIRI, with both protective and deleterious effects described [31, 32, 33].In line with this complexity, hepatocyte‐targeted FOXO1 silencing supports a functional requirement for hepatocellular FOXO1 in sustaining SAA induction and downstream enrichment of FPR2^+^ macrophages, implicating this cascade in the pericentral predominance of injury in HIRI. Clinically, spatial colocalization of FOXO1 and SAA in post‐transplant liver tissue, together with elevated circulating SAA and its association with ALT and AST, suggests that SAA may serve as an adjunct indicator of hepatocellular injury burden after transplantation.

These mechanistic insights also inform therapeutic prioritization. Strategies targeting oxidative stress, inflammation, and thrombosis in LT‐induced HIRI remain largely preclinical, underscoring the limited availability of clinically tractable options [34, 35, 36, 37]. In this context, our data support SAA‐directed intervention as a mechanism‐based strategy that directly targets a hepatocyte‐to‐macrophage inflammatory cascade implicated in pericentral injury amplification. However, the clinical translatability of SAA‐neutralizing antibodies remains unvalidated to date. Amilo‐5MER is a peptide that inhibits SAA hexamer assembly and thereby suppresses SAA bioactivity. This mechanism has been validated in inflammatory diseases, including rheumatoid arthritis, inflammatory bowel disease, and multiple sclerosis [19]. Notably, a recent clinical trial has completed the safety assessment of Amilo‐5MER (NCT05857215). Together with the graft‐protective effects observed in our study, these attributes strengthen the rationale for prioritizing Amilo‐5MER as a lead candidate for SAA‐targeted graft protection.

In conclusion, our multimodal atlas identifies pericentral hepatocyte stress and macrophage‐mediated amplification as an important determinant of lobularly patterned injury in LT‐induced HIRI. We define a FOXO1‐driven SAA/FPR2 axis coupling hepatocyte stress to macrophage recruitment, and show that Amilo‐5MER suppresses SAA bioactivity and attenuates liver injury in LT‐induced HIRI. Limitations of this study include the multifactorial nature of zonal vulnerability during LT‐associated HIRI. Although our data support a macrophage‐driven amplification program centered on the FOXO1/SAA/FPR2 axis, we cannot quantitatively disentangle its contribution from concurrent physiological and injury determinants, including hypoxia and perfusion gradients, metabolic stress, and loss of cell‐cell contact. Moreover, our macrophage‐focused experiments were designed around FPR2 expression and did not systematically interrogate the full spectrum of macrophage subsets and states that may contribute to LT‐induced HIRI. Future studies integrating higher‐resolution macrophage state mapping with subset‐selective perturbations will be important to delineate complementary or opposing macrophage programs during injury and repair. Finally, biomarker analyses were performed in a modest clinical cohort, warranting validation of serum SAA in larger independent datasets with multivariable adjustment for key confounders. Future work should prioritize clinically proximate testing of SAA‐targeted strategies, particularly in ex vivo machine perfusion settings, and should evaluate whether donor‐ and graft‐specific features can guide patient stratification and rational combination with interventions targeting complementary injury pathways.

## 4 Experimental Section

### 4.1 Clinical Specimen Collection and Data Analysis

This study analyzed donor liver tissue samples collected during the pre‐ and post‐reperfusion phases of liver transplantation surgery. The specimens were collected by the Department of Hepatic Surgery and Liver Transplantation Center of the Third Affiliated Hospital of Sun Yat‐sen University between May 2022 and May 2025. Preperfusion samples were acquired within 2 h of organ procurement but prior to cold perfusion, whereas postreperfusion samples were collected within 2 h following portal revascularization of the graft. In addition, ten paired pre and postoperative blood samples were collected from an independent set of cases in 2025. Whole blood was centrifuged at 800 × g for 10 min to isolate serum for subsequent assays. Postoperative liver function markers, including the serum alanine aminotransferase (ALT) and aspartate aminotransferase (AST) levels from post‐operative days 1–7, were obtained retrospectively from the hospital's electronic medical records system. This study was approved by the Clinical Research Ethics Committee of the Third Affiliated Hospital of Sun Yat‐sen University (RG2024‐117‐02). Written informed consent was secured from all participants prior to their involvement in the study.

### 4.2 Animals

Wild‐type C57BL/6J mice (male) used in this study were purchased from Wukong Biotechnology (Jiangsu, China). All experimental animals were housed under specific pathogen‐free (SPF) conditions at the animal facility of Gennio Biotechnology Co., Ltd's (Guangzhou, China). All mice were housed under controlled environmental conditions, including a 12‐h light/dark cycle, ambient temperature between 20°C and 25°C, and relative humidity at 30%–70%. The experimental protocol received ethical approval from the Institutional Animal Care and Use Committee of Jennio Biotech Co., Ltd (#JENNIO‐IACUC‐2025‐A025), and all procedures were performed in accordance with Chinese legislation governing the use of experimental animals.

The hepatic ischemia‐reperfusion injury (HIRI) model was constructed by intraperitoneally anesthetizing mice with sodium pentobarbital (50 mg/kg), followed by blocking the blood supply to the left and median liver lobes for 90 min using atraumatic vascular clamps, and then releasing the clamps to initiate reperfusion. The sham group underwent laparotomy without vascular occlusion. After 24 h postreperfusion, blood and liver tissue samples were collected for subsequent examinations.

For in vivo SAA neutralization, animals were intravenously treated with goat anti‐mouse SAA polyclonal antibody (5 µg/mouse; AF2948, R&D Systems, USA), with normal goat IgG serving as the isotype control. For in vivo SAA inhibition, Amilo‐5MER (10 mg/kg; HY‐P10935A, MedChemExpress, USA) was dissolved in a vehicle containing 10% DMSO (D5879, Sigma‐Aldrich, USA) and 20% sulfobutylether‐β‐cyclodextrin (SBE‐β‐CD; HY‐17031, MedChemExpress, USA), then administered intraperitoneally. The control group received an equivalent volume of the vehicle. All treatments were performed 2 h before ischemia induction, as per the manufacturers' protocols. The number of mice in each experiment is indicated in the figure legends.

### 4.3 Evaluation of Liver Injury

The serum levels of ALT and AST in mice were measured via commercial kits (Beijing Boxbio Science & Technology Co.,Ltd., China). Liver tissues were fixed in 4% paraformaldehyde (PFA), embedded in paraffin, and sectioned to a thickness of 4 µm, followed by hematoxylin and eosin (H&E) staining. Liver injury was assessed using the Suzuki scoring system, which evaluates three histological features: sinusoidal congestion, hepatocyte necrosis, and ballooning degeneration. Each feature was scored on a scale of 0–4. Five random fields were examined per sample, and the final Suzuki score was calculated as the average of these fields.

### 4.4 Terminal‐Deoxynucleotidyl Transferase Mediated Nick End Labeling (TUNEL) Assay

TUNEL assay was performed using the One‐Step TUNEL Apoptosis Assay Kit (C1088, Beyotime Biotechnology, China) following the manufacturer's instructions. Briefly, after washing twice with PBS, the samples were fixed with 4% PFA for 20 min at room temperature (RT) and permeabilized with 0.3% Triton X‐100 for 10 min. Subsequently, the TUNEL reaction mixture was incubated at 37°C for 1 h in the dark. After counterstaining with DAPI, apoptotic cells were identified by TUNEL‐positive staining using a fluorescence microscope (Nikon, Japan) and quantified by counting positive cells in five randomly selected fields per section. TUNEL‐positive rate was calculated as the number of TUNEL‐positive cells relative to the total number of DAPI‐stained cells × 100% [38].

### 4.5 Immunohistochemical (IHC) Staining

Staining procedures were performed on 4‐µm paraffin‐embedded liver sections following deparaffinization in xylene and graded ethanol rehydration. Target antigen exposure was achieved through heat‐mediated retrieval using antibody‐specific buffer conditions. After blocking with normal goat serum for 30 min at RT, sections were incubated overnight at 4°C with primary antibodies against FOXO1 (1:200, Abcam, Cambridge, UK). Then, secondary antibody incubation and DAB coloration were performed using the Dako REAL EnVision Detection System (K5007, Copenhagen, Denmark). Finally, sections underwent hematoxylin nuclear counterstaining followed by standard dehydration and mounting procedures. Staining results were semi‐quantitatively assessed by two blind investigators evaluating five random fields per sample, with IHC staining scores calculated as the product of intensity (1: negative, 2: weak, 3: moderate, 4: strong) and extent scores (1: 0%–25%, 2: 25%–50%, 3: 50%–75%, 4: 75%–100% positive cells).

### 4.6 Immunofluorescence (IF) Staining

Paraffin‐embedded tissue sections were deparaffinized through xylene immersion and progressively hydrated. Following a brief rinse in distilled water, heat‐induced epitope retrieval was performed using sodium citrate buffer (pH 6.0) with three cycles of microwave irradiation (4 min each). After cooling naturally to RT, sections were permeabilized with 0.5% Triton X‐100 for 1 h and treated with 3% bovine serum albumin (BSA) to minimize nonspecific antibody interactions. Subsequently, samples were incubated at 4°C overnight with primary antibodies against: GS (1:400, Abcam), E‐cadherin (1:500, R&D), γ‐H2A.X (1:200, Abcam), FPR2 (1:200, Abcam), F4/80 (1:200, Abcam), CD68 (1:200, Affinity), FOXO1 (1:100, Cell Signaling Technology), SAA (1:200, Proteintech). After washing, samples were exposed to fluorophore‐conjugated secondary antibodies (Invitrogen) for 1 h at RT under light‐protected conditions. Nuclei were labeled with DAPI for 5 min at RT. Image acquisition and analysis were conducted using a confocal microscope (Zeiss 880, Nikon Instruments, Melville, New York, USA) manufacturer‐provided software.

### 4.7 Enzyme‐Linked Immunosorbent Assay (ELISA)

The concentrations of SAA1 were quantified using commercially available mouse ELISA kits (for cell culture supernatants; MM‐46455M2, Jiangsu Meimian Industrial Co., Ltd, China), and human ELISA kit (for participants' plasma; BDEL0724‐96T, Biodragon, China), following the respective manufacturer's guideline.

### 4.8 Isolation and Culture of Mouse Primary Hepatocytes

Primary mouse hepatocytes were isolated from C57BL/6J mice using an established protocol [39]. In brief, under sterile conditions, liver digestion was initiated by portal vein perfusion with prewarmed Hank's balanced salt solution (HBSS) containing 0.5 mg/mL type IV collagenase (C4‐28‐100MG, Sigma‐Aldrich, USA). After complete digestion, the liver capsule was gently torn open using curved forceps, and the cell suspension was filtered through a 70‐µm strainer using a sterile Pasteur pipette. Parenchymal cells were pelleted through three cycles of centrifugation (50 × g, 5 min, 4°C) to enrich hepatocytes. The resulting cell pellet was resuspended in 20 mL complete medium and further purified by density gradient centrifugation on 90% Percoll (200 × g, 10 min, 4°C). The purified hepatocytes were then collected from the pellet, maintained in DMEM/F‐12 medium supplemented with 1% ITS‐G (S450J7, BasalMedia, China), and cultured in a cell dish or chambers (Jet Biofil, China; NEST Biotechnology, China).

### 4.9 In Vitro Hypoxia Reoxygenation (H/R) Model

To mimic HIRI in vitro, we established an H/R model in primary mouse hepatocytes. Briefly, cells were maintained in serum‐free basal medium during 6 h of hypoxic conditions (1% O_2_, 5% CO_2_, and 94% N_2_), followed by 2 h of reoxygenation in complete medium under normoxic conditions (21% O_2_, 5% CO_2_).

### 4.10 Isolation of Intrahepatic Macrophages

Primary mouse liver macrophages were isolated through a sequential enzymatic and mechanical dissociation process as previously described [40]. Initial perfusion was performed through the portal vein using calcium/magnesium‐free HBSS with 0.5 mM EGTA (ST068, Beyotime, China), followed by collagenase IV digestion. The enzymatically treated livers were then carefully dissociated using forceps to create single‐cell suspensions. After passing through a 70 µm cell strainer, hepatocytes were pelleted through low‐speed centrifugation (50 × g, 5 min, 4°C). The supernatant containing non‐parenchymal cells was further concentrated by centrifugation (300 × g, 5 min, 4°C). The collected cell suspension was resuspended in RPMI‐1640 with 10% FBS and separated on a 25%/50% Percoll gradient (1200 × g, 30 min, 4°C, no brake). Macrophages were collected from the interphase layer for subsequent fluorescence‐activated cell sorting.

### 4.11 Fluorescence‐Activated Cell Sorting (FACS)

For sorting of macrophage subsets in the mouse liver, intrahepatic macrophages were isolated from the mouse liver as described above and labeled using the Zombie Aqua Fixable Viability Kit (423101, BioLegend) to discern live cells. Subsequently, cells were stained with APC/Cyanine7 antimouse CD45 Antibody (103116, Biolegend), PerCP/Cyanine5.5 antimouse/human CD11b Antibody (101227, Biolegend), APC antimouse F4/80 Antibody (123115, Biolegend), and FITC anti‐mouse FPRL1/FPR2 Antibody (NLS1878F, NOVUS) at a 1:200 dilution. FPR2‐specific populations were identified following spectral compensation, with gating thresholds established using fluorescence‐minus‐one (FMO) controls.

Pericentral (PC) and periportal (PP) hepatocytes sorting was conducted as previously described [41, 42]. Briefly, primary mouse hepatocytes were isolated according to the above illustration and resuspended in ice‐cold PBS at a concentration of 1 × 10^6^ cells per 100 µL. To discriminate live cells, the Zombie Green Fixable Viability Kit (423111, Biolegend) was added. Subsequently, cells were stained with APC/Cyanine7 anti‐mouse CD45 Antibody (103116, Biolegend), Brilliant Violet 605 anti‐mouse CD31 Antibody (102427, Biolegend), APC anti‐mouse CD73 Antibody (127210, Biolegend), and PE anti‐mouse/human CD324 (E‐Cadherin) Antibody (147304, Biolegend) at a 1:200 dilution.

TruStain FcX PLUS (antimouse CD16/32) Antibody (156603, BioLegend) was included in a dilution of 1:50. After washing, flow cytometry and cell sorting were performed on a CytoFLEX SRT flow cytometer (Beckman Coulter, Brea, CA, USA) with FlowJo software (BD Biosciences, USA).

### 4.12 Macrophage Adoptive Transfer and Fluorescent Dye‐Labeled Tracking

Forty‐eight hours prior to macrophage transfer, recipient mice received a single intravenous administration of 200 µL neutral clodronate liposomes (CLLs; F70101C‐N, FormuMax Scientific, USA) to achieve systemic macrophage depletion. After this pretreatment, the standardized HIRI model was established. During the reperfusion phase, freshly purified FPR2‐high or FPR2‐low hepatic macrophages from donor mice were delivered into recipient spleens using 30‐gauge insulin needles to ensure engraftment.

For macrophage tracking in the HIRI model, flow cytometry‐sorted intrahepatic macrophages were labeled with lipophilic fluorescent markers DiR (1,1‐dioctadecy‐3,3,3,3‐tetramethy‐lindotricarbocyanine iodide, Thermo Fisher Scientific, USA). In brief, cells were incubated with 1 µM dye solution for 10 min at RT in darkness, followed by quenching with 10% BSA/PBS and centrifugation. PBS‐treated cells served as controls. The labeled macrophages were administered via intrasplenic injection during reperfusion, with in‐vivo distribution assessed 24 h post‐transfer using Bruker Small Animal Optical Imaging System (In Vivo Xtreme II; Billerica, MA). Subsequently, ex vivo evaluation involved harvesting major organs (heart, lungs, liver, spleen, and kidneys) for fluorescence imaging and staining quantification of macrophage localization patterns.

### 4.13 Cell‐Migration Assay

FPR2‐high and FPR2‐low macrophages were isolated via FACS from the mouse HIRI model, and then seeded into the upper chamber of a transwell system at a density of 2.0 × 10^5^ cells per well. The lower chamber was plated with 4.0 × 10^5^ untreated primary mouse hepatocytes. Three experimental conditions were established: control, H/R, and H/R treated with Amilo‐5MER. After co‐culture, the transwell insert was removed, and the adherent liver macrophages in the lower chamber were quantified by IF staining for F4/80 (1:200, Abcam).

### 4.14 RNA Extraction and Quantitative Real‐Time PCR (qRT‐PCR)

Total RNA was extracted using TRIzol Reagent (Invitrogen) following the manufacturer's instructions [43]. The extracted RNA was reverse‐transcribed into cDNA using the Evo M‐MLV Reverse Transcription Kit (Accurate Biotechnology, China). Then, qRT‐PCR was performed using the SYBR qPCR SuperMix Plus (E096‐01B, Novoprotein, Shanghai, China) according to the manufacturer's protocol. *β‐actin* or *Gapdh* was used as the internal reference gene. The specific primer sequences are listed in Table S1.

### 4.15 Cytoplasmic and Nuclear Protein Separation Assay

Primary mouse hepatocytes (1 × 10^7^ cells) were harvested by trypsinization, washed with ice‐cold PBS, and subjected to subcellular fractionation using the NE‐PER Nuclear and Cytoplasmic Extraction Kit (Thermo Fisher Scientific, USA) according to the manufacturer's protocol. All extraction procedures were carried out on ice to preserve protein stability. Protein concentrations were determined by the BCA protein assay before Western blot analysis. Histone H3 and β‐actin were used as internal controls for nuclear and cytoplasmic fractions, respectively.

### 4.16 Western Blotting Assays

Western blot analysis was performed to determine target protein expression levels according to previously described protocols [39]. Briefly, total proteins were extracted using RIPA lysis buffer (L‐7103, Biolinkedin, Shanghai, China). Then, protein samples were separated by 12% SDS‐PAGE and subsequently transferred onto 0.45 µm PVDF membranes (Merck Millipore, USA). After blocking with 5% skim milk for 1 h at RT, the membranes were incubated overnight at 4°C with primary antibodies against: FOXO1 (1:1000; Cell Signaling Technology, USA), SAA (1:1000; Proteintech, China), Histone H3 (1:2000; Cell Signaling Technology, USA), β‐actin (1:5000; Immunoway, USA). Following three washes with Tris‐buffered saline with Tween 20 (TBST), the membranes were probed with HRP‐conjugated secondary antibodies for 1 h at RT. Immunoreactive bands were visualized using an enhanced chemiluminescence substrate (ECL; Merck KGaA, Germany) and imaged with a FluorChem system (ProteinSimple, USA).

### 4.17 Plasmids, Transfection, Infection

The pCMV6‐FLAG‐MCS‐Neo and pCMV6‐Foxo1(mouse)‐3×HA‐Neo plasmids were purchased from Miaoling Bio (Wuhan, China). siRNAs targeting *Foxo1* were designed and synthesized by GenePharma (Shanghai, China). The transfection of plasmids and siRNAs was performed using jetPRIME (Polyplus‐transfection, China) according to the manufacturer's procedure. The sequences of siRNAs are listed in Table S1.

### 4.18 Animal Adeno‐Associated Virus‐8 (AAV8) Injection and Processing

Hepatocyte‐specific *Foxo1* and *Saa1* knockdown in mice was achieved via tail vein injection of shRNA‐carrying adeno‐associated virus serotype 8 (AAV8) bearing thyroxine‐binding globulin (TBG) promoter. Macrophage‐specific *Fpr2* knockdown was achieved via tail vein injection of shRNA‐carrying AAV8 bearing F4/80 promoter. The recombinant AAV8 vectors were purchased from PUZONGene Technology (guangzhou). The control group received an equal dose of AAV8 carrying a nontargeting shRNA sequence. After 4 weeks, the HIRI model was established in the indicated mice and sacrificed later.

### 4.19 Chromatin Immunoprecipitation (ChIP) qPCR

The ChIP assays were performed using a commercial kit (P2078, Beyotime Biotechnology, China) following the manufacturer's protocol. In brief, 1 × 10^7^ primary mouse hepatocytes were fixed with 1% formaldehyde at 37°C for 10 min, followed by glycine quenching. Postwashing twice with PBS + 1 mM PMSF, cell lysis was performed in SDS buffer + 1 mM PMSF (10 min, ice). Chromatin was then sonicated (10% intensity, 5‐s pulses), centrifuged (14 000 g, 5 min, 4°C), and the resulting supernatant diluted in 2 mL ChIP Dilution Buffer + 1 mM PMSF. 10% aliquot of the lysate was saved as input control, while the remaining lysate was incubated overnight at 4°C with either anti‐FOXO1 antibody (Cell Signaling Technology, USA) or control IgG. Immunoprecipitated DNA was purified and analyzed by qPCR using *Saa1* promoter‐specific primers (Table S1), with mouse *Gapdh* serving as the negative control

### 4.20 RNA‐Seq Analysis

Total RNA was extracted from human liver tissues using standard protocols. After extracting the total RNA, the samples were subjected to agarose gel electrophoresis, Nanodrop quality assessment, and quantification. For mRNA enrichment, oligo (dT) magnetic beads were used. RNA‐Seq libraries were constructed using the KAPA Stranded RNA‐Seq Library Prep Kit (Roche) that involved RNA fragmentation, randomly primed reverse transcription to generate first‐strand cDNA, synthesis of second‐strand cDNA using dUTP, end repair of double‐stranded cDNA with A‐tailing, and ligation of the Illumina adapter for sequencing. The final library was amplified using PCR. The constructed libraries were quality checked using an Agilent 2100 Bioanalyzer, and library quantification was performed using qPCR. Sequencing was performed on an Illumina NovaSeq 6000 sequencer. Raw reads in FASTQ format were pre‐processed using fastp (v0.24.0) to remove low‐quality reads and adapter sequences [44]. Reference genome and gene model annotation files were downloaded from the Ensembl website directly [45]. An index of the reference genome was built using Hisat2 (v2.2.1), and paired‐end clean reads were aligned to the reference genome [46]. For quantification of gene expression, read pairs were excluded that have their two ends mapping to different chromosomes, mapping to the same chromosome but on different strands, or do not have both ends aligned. Only the reads aligned to exons, and with the mapping quality score larger than 10 were counted using the featureCounts (v2.0.8) package [47]. Differential gene expression analysis was conducted using the DESeq2 R package (1.46.0), which models count data based on a negative binomial distribution [48]. *p*‐values were adjusted for multiple testing using the Benjamini–Hochberg procedure to control the false discovery rate.

### 4.21 Label‐Free Quantitative Proteomics

Total protein was extracted from liver tissues and subjected to enzymatic digestion. The resulting peptides were separated and analyzed by Shu Pu (Shanghai) Biotechnologies LLC (Aksomics, Shanghai, China). using a nano‐UPLC (EASY‐nLC1200) system coupled to a Q Exactive mass spectrometer (Thermo Fisher Scientific), with a 120‐min gradient per sample. Raw MS files were processed with MaxQuant (v1.5.6.0) [49]. The protein sequence database (Uniprot_organism_2016_09) was downloaded from UNIPROT. For differential protein detection, proteins quantified in at least two samples of each condition were kept for further analysis. We performed differential detection analysis using DEqMS (v1.24.0) package [50, 51].

### 4.22 ATAC‐Seq Analysis

Human liver tissues were processed into single‐cell suspensions, and cell viability was assessed using Trypan Blue exclusion staining. Nuclei were isolated, and chromatin was tagmented using Tn5 transposase. The tagmented DNA fragments were purified using the QIAquick PCR Purification Kit (Qiagen, #28106) according to the manufacturer's protocol. Purified DNA was then PCR‐amplified to construct sequencing libraries. Size selection was performed by gel electrophoresis to remove primer dimers and large DNA fragments. Libraries were clustered on a flow cell and sequenced using an Illumina sequencing platform. Fastp v0.24.0 was used to remove adapters and low‐quality reads [44]. Paired‐end reads were then mapped to the human genome (Ensembl GRCh38) using Bowtie2 v2.5.4 [52]. After alignment, unique aligned reads were filtered with map quality and duplicates (Picard MarkDuplicates) [53]. For ATAC‐seq analysis, reads mapped to MT chromosome and reads overlapping the ENCODE hg38 functional genomics regions blacklist were also removed to improve the quality of the retained fragments [54]. To correct for the fact that the Tn5 transposase binds as a dimer and inserts two adapters in the Tn5 tagmentation step, all positive‐strand reads were shifted 4 bp downstream, and all negative‐strand reads were shifted 5 bp upstream to center the reads on the transposase binding event. Peaks were then called individually for each replicate using macs3 v3.0.2 [55]. Differential analysis of peaks in ATAC‐seq was performed using Diffbind v3.16.0 [56]. Peak annotation and genomic feature association were conducted using the ChIPseeker v1.42.0 [57].

### 4.23 10x Genomics Chromium Library Construction and Sequencing

Library synthesis was performed following the Chromium Next GEM Single Cell 3ʹ Reagent Kits v3 protocol (10x Genomics). Briefly, cell suspensions were adjusted to 1000 cells/µL and loaded onto the Chromium Controller to generate single‐cell Gel Beads‐In‐Emulsions (GEMs). Within each GEM, individual cells were isolated alongside gel beads coated with unique primers containing 10x cell barcodes, unique molecular identifiers (UMIs), and poly(dT) sequences. Reverse transcription was conducted in GEMs using a Veriti 96‐well thermal cycler (Thermo Fisher Scientific, Waltham, MA, USA). Subsequently, cDNA libraries underwent amplification, fragmentation, end repair, A‐tailing, adaptor ligation, sample index PCR, and final purification using SPRIselect beads. The libraries were then sequenced on the Illumina NovaSeq 6000 platform to a depth of approximately 500 million reads per library with a 2 × 150 read length.

### 4.24 10x Genomics Visium HD Library Construction, Sequencing, and Analysis

To analyze the tissue sample by using a formalin‐fixed paraffin‐embedded (FFPE) Visium CytAssist spatial gene expression assay (10x Genomics). After taking 5 paraffin sections with a thickness of 5 um, the integrity of RNA was detected by Agilent 2100 bioanalyzer, and DV200 ≥ 30% was considered satisfactory. VISIUM CytAssist spatial gene expression slides and reagent kits were used according to the manufacturer's instructions (10x Genomics). We placed FFPE tissue sections on plain glass slides for deparaffinization, H&E staining, and imaging following the Visium HD FFPE Tissue Preparation Handbook (CG000684). Probe hybridization, probe ligation, slide preparation, probe release, extension, library construction, and sequencing followed the Visium HD Spatial Gene Expression Reagent Kits User Guide (CG000685). Sequencing was performed on an Illumina NovaSeq 6000 with paired‐end reads (43 cycles Read 1, 10 cycles i7, 10 cycles i5, 50 cycles Read 2). Space Ranger v3.0 (10x Genomics) is used to map FASTQ files to reference genomes, examine tissue sections, align sequencing data to microscope images and CytAssist images, and output gene barcode matrices for further analysis. These matrices were converted into Seurat objects using the Seurat v5.1.0 package in R, with 8‐µm binned data employed for analysis following official Visium HD tutorials [58].

### 4.25 Quality Control of scRNA‐Seq Data

Raw scRNA‐seq data were processed using 10× Genomics Cell Ranger (v7.1.0) with default parameters for demultiplexing, alignment to mouse and human reference genomes, and UMI counting (by using the *cellranger count* function). The UMI count matrixes were transformed into Seurat objects using the R package Seurat v4.4.0 [58]. Cells expressing fewer than 200 genes or with mitochondrial gene content exceeding 10% of total UMIs were excluded. Doublets were identified and removed using DoubletFinder (v2.0.3) with the 92.4 percentile as the cutoff [59]. Additionally, cells with UMI counts over 40 000 or detecting more than 6000 genes in mice or 5000 genes in humans were filtered out. Filtered matrices were then merged for downstream analysis.

### 4.26 ScRNA‐Seq Data Processing and Visualization

Each dataset was normalized using the *SCTransform* function in Seurat v4.4.0, and the heterogeneity associated with mitochondrial contamination was regressed out [58]. Features and anchors for downstream integration were selected according to the SCTransform pipeline. Datasets were integrated using canonical correlation analysis with *FindIntegrationAnchors* and *IntegrateData* functions. Principal component analysis was performed with *RunPCA* function, and appropriate principal components were selected for subsequent analysis. Dimensionality reduction was achieved using *RunUMAP* function, and a shared nearest neighbor graph was constructed with *FindNeighbors* function. Clustering was conducted using *FindClusters* function. Putative cross‐lineage multiplets were removed based on the coexpression of canonical markers. Pathway signature module scores were assessed with *AddModuleScore* function, utilizing functional signatures from the Molecular Signatures Database (MSigDB) collection [60, 61, 62]. Enrichment analysis to investigate biological states or functional differences among cell types was performed using Metascape (v3.5) with default parameters [63]. Single‐cell regulatory networks for each subcluster were constructed using the decoupleR package (v2.9.7) with default parameters [64]. Prediction of the transcriptional factor of SAA1 was conducted using TF‐Target Finder (TFTF), and the binding sites were obtained by JASPAR database [65, 66]. Visualization was carried out using SCP (v0.5.1) (https://github.com/zhanghao‐njmu/SCP).

### 4.27 Prediction of Ligand‒Receptor Interactions

To identify significant signaling molecules among liver microenvironment populations, we utilized CellChat (v2.1.2) [67]. The normalized counts of merged liver samples were loaded into CellChat, and the data were pre‐processed using the *identifyOverExpressedGenes, indentifyOverExpressedInteractions* functions based on the “CellChatDB.mouse” and “CellChatDB.human” databases. The *computeCommunProb*, *computeCommunProbPathway* and *aggregeNet* functions were applied in a sequential manner to infer significant ligand‐receptor pairs across cell types.

### 4.28 Statistical Analysis

All statistical analyses were performed using GraphPad Prism (version 10.0) and R software (version 4.2.1). Data are presented as mean ± standard error of the mean (SEM) based on at least three biological replicates, with the number of replicates (n) indicated for each experimental condition. The primary outcomes in this study were continuous variables unless otherwise indicated. Normality and homogeneity of variances were verified. Comparisons between two groups were conducted using unpaired two‐tailed Student's T‐tests. For comparisons involving three or more independent groups, one‐way ANOVA with Tukey's post hoc tests was applied for experiments with a single independent variable, whereas two‐way ANOVA followed by Sidak's post hoc tests was used for factorial designs involving two independent variables. Pearson's correlation coefficient was employed to assess the relationship between two continuous variables. All statistical tests were two‐sided, with *p* < 0.05 considered statistically significant.

## Author Contributions

FZ and YL initiated the project; FZ, RL, TW, XY, and YL designed experiments; FZ, TW, J‐H Z, ZW, XL, QY, CX, J‐B Z, HC, and JX carried out experiments and analyzed the experimental data; YL analyzed the single‐cell sequencing data; JY, HL, SY, and YY provided human hepatic samples and clinical information; RL verified statistics; YL, FZ, XY, RL, and TW wrote the paper. QZ, YY, JZ, and YZ reviewed the manuscript and participated in the interpretation of data.

## Conflicts of Interest

The authors declare no conflicts of interest.

## Supporting information




**Supporting file**: advs75074‐sup‐0001‐SuppMat.docx

**Supporting file**: advs75074‐sup‐0001‐TableS1.docx

## Data Availability

The datasets analyzed during the current study are available from the corresponding author on reasonable request. This study did not use any unique codes, and all analyses were performed in R and Python using standard protocols from previously published packages.
