# Supplementary material for: SAA/FPR2 Signaling Between Pericentral Hepatocytes and Macrophages Exacerbates Zonated Liver Transplant Injury
Source: Adv Sci (Weinh). 2026 Mar 29;13(34):e22891. doi: 10.1002/advs.202522891 (PMC13285152; doi:10.1002/advs.202522891)
Supplement: Supplementary file 1 — Supporting file: advs75074‐sup‐0001‐SuppMat.docx [file ADVS-13-e22891-s002.docx]

# **Supporting Information**

**SAA/FPR2 Signaling between Pericentral Hepatocytes and Macrophages Exacerbates Zonated Liver Transplant Injury**

Feng Zhang^1, 2, 3, #^, Rong Li^2, #^, Tingting Wang^1, 2, #^, Jianhao Zhang^1, 2, #^, Zhengqi Wu^1, 2^, Qiang You^1, 2^, Xuying Liu^1, 2^, Cuicui Xiao^4^, Jiebin Zhang^1, 2^, Haitian Chen^1, 2^, Jiaqi Xiao^1, 2^, Jia Yao^1, 2^, Jun Zheng^1, 2^, Yingcai Zhang^1, 2, 5^, Hua Li^1, 2^, Shuhong Yi^1, 2^, Yang Yang^1, 2, *^, Qi Zhang^3, *^, Xiaofeng Yuan^6, *^, Yasong Liu^1, 2, *^

**Supplement Figure Legends**

**
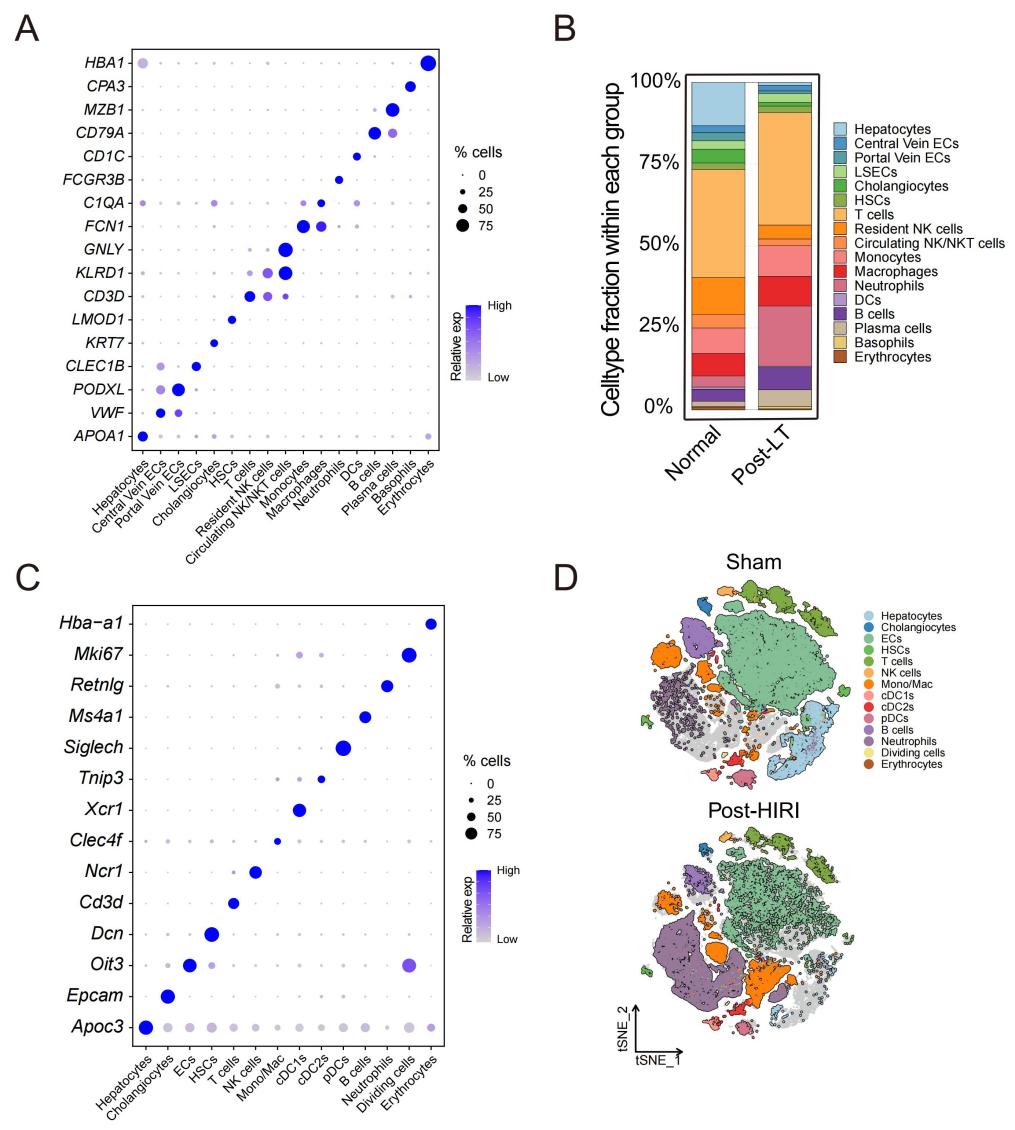
**

**Supplementary Figure 1. Cell-type marker expression and composition in human and mouse livers.**

1. Violin plots showing the expression levels of representative cell type-specific marker genes for corresponding cell types in human donor livers. (B) Stacked bar charts showing the relative proportions of major hepatic cell types in normal versus post-LT groups. (C) Violin plots showing the expression levels of representative cell type-specific marker genes for corresponding cell types in mouse livers. (D) Corresponding cell type distributions are shown across different groups.


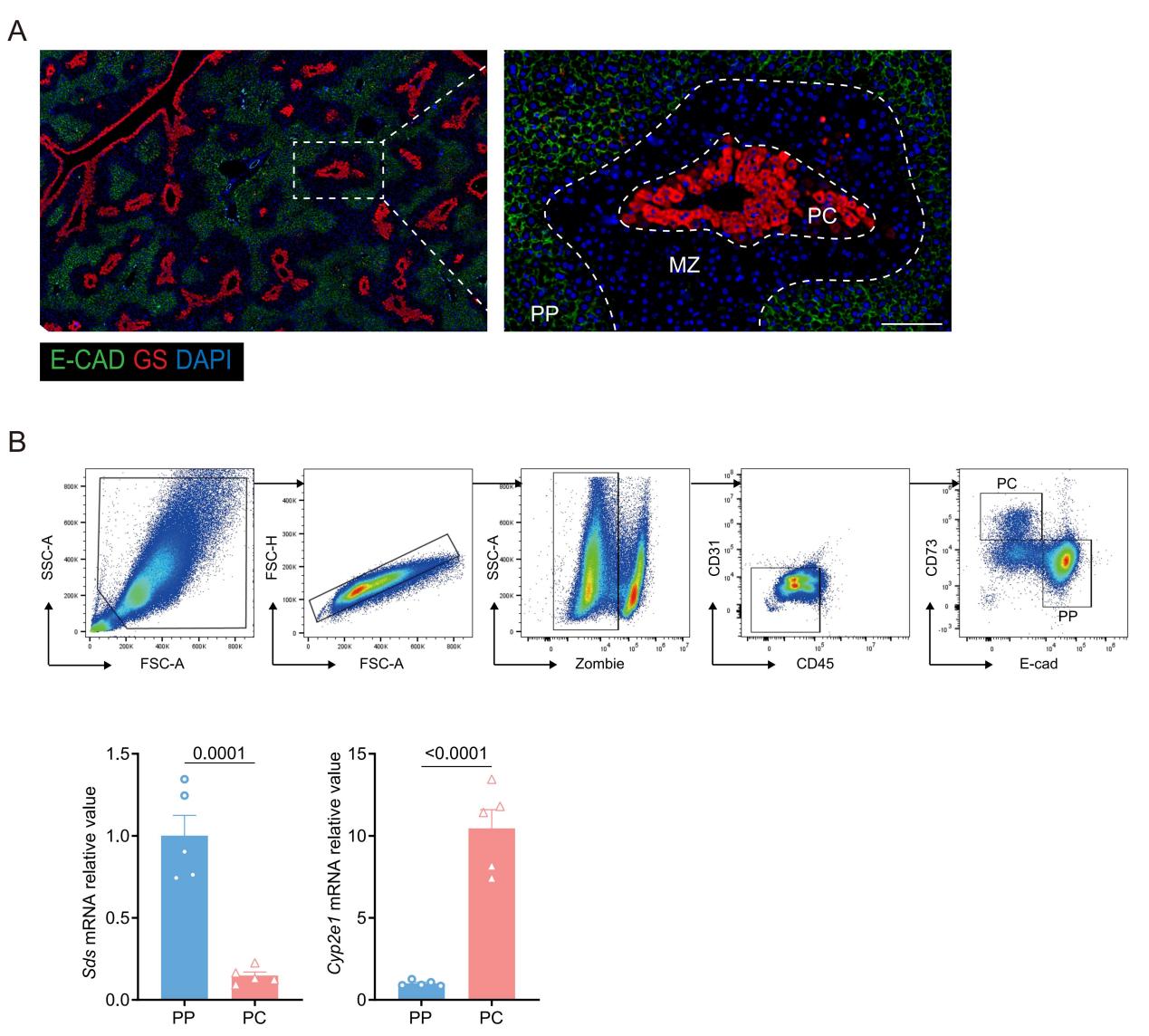


**Supplementary Figure 2. Zonation markers and isolation of periportal vs pericentral hepatocytes in mouse liver**

1. Immunofluorescence (IF) staining delineating zonated hepatocytes in mouse livers: E-cadherin (E-CAD, green) marks periportal (PP) hepatocytes, glutamine synthetase (GS, red) marks pericentral (PC) hepatocytes, and cells located between PP and PC correspond to midzonal (MZ) hepatocytes. Scale bar, 100 μm. (B) Flow cytometry sorting strategy for PP and PC hepatocytes (upper), accompanied by qRT-PCR validation of *Sds* and *Cyp2e1* expression (lower). n = 5, per group. P < 0.05 was considered significant.


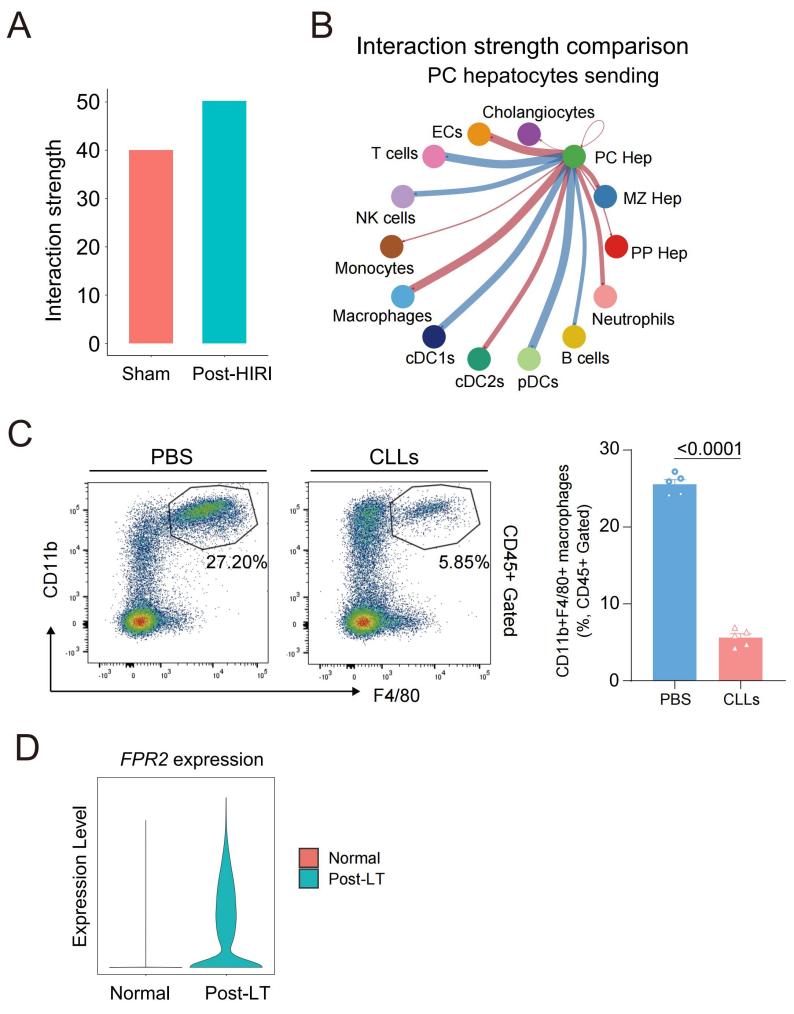


**Supplementary Figure 3. Enhanced hepatocyte–macrophage signaling and FPR2 expression in macrophages**

1. Intercellular interaction strength within the hepatic microenvironment in Sham and post-HIRI mouse livers. (B) Circle plot illustrating differential intercellular signaling interactions sent by PC hepatocytes in mouse. Line color indicates signaling interaction change in post-LT donor livers (red, upregulation; blue, downregulation). Line thickness represents changes in interaction strength. (C) Flow cytometry confirms hepatic macrophage depletion by clodronate liposomes (CLLs). Representative CD11b versus F4/80 plots (left) and quantification of CD11b⁺F4/80⁺ cells (right) among CD45⁺ liver leukocytes 48 h after PBS or CLLs injection (n = 5, per group). (D) ScRNA-seq visualization of *FPR2* expression in macrophages from normal versus post-LT human donor livers. P < 0.05 was considered significant.


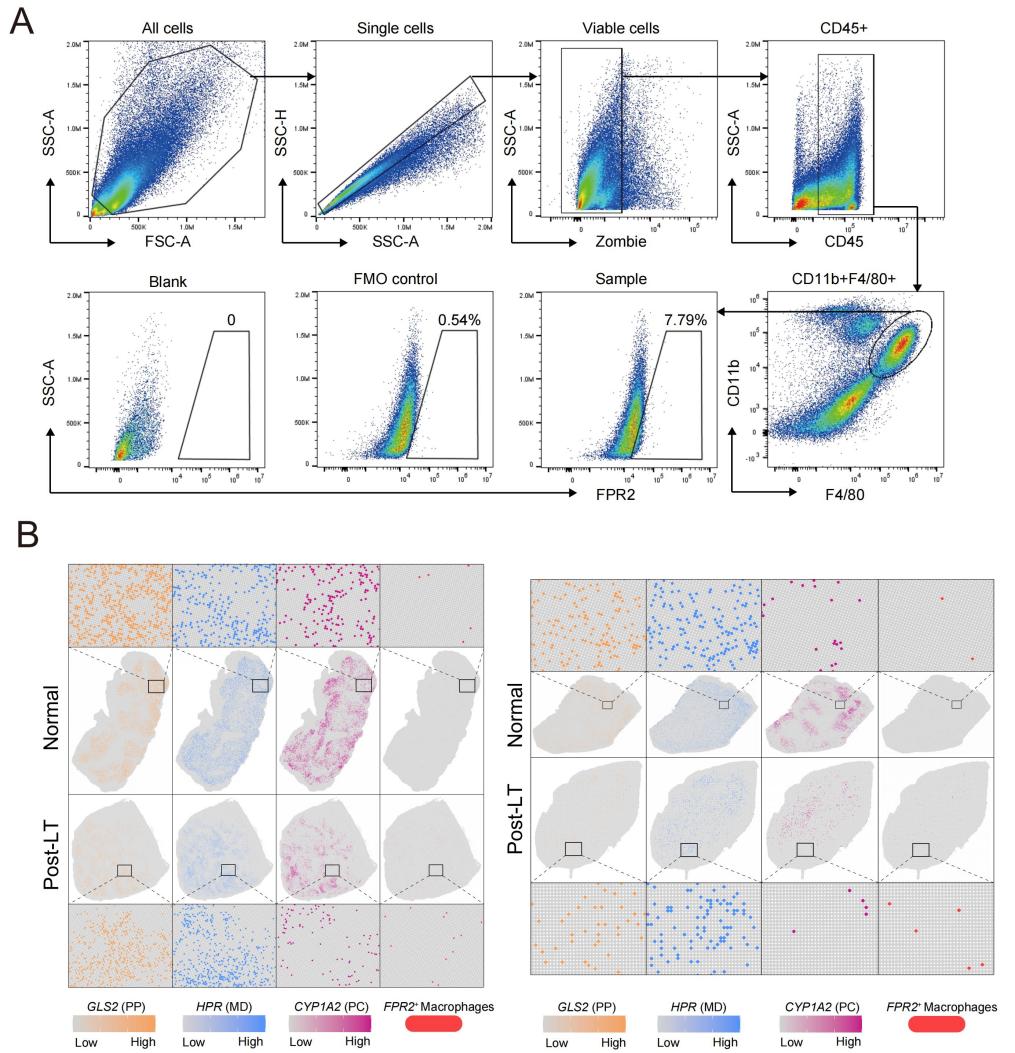


**Supplementary Figure 4. Flow cytometry gating and spatial zonation mapping of FPR2^+^ macrophages.**

1. The gating strategy and FMO control for flow cytometry in Figure 3H. (B) Spatial transcriptomic maps showing the expression of *GLS2* (PP), *HPR* (MZ), and *CYP1A2* (PC) in normal and post-LT donor livers, together with the spatial distribution of FPR2^+^ macrophages. Representative regions were magnified to highlight areas enriched for zonation markers.


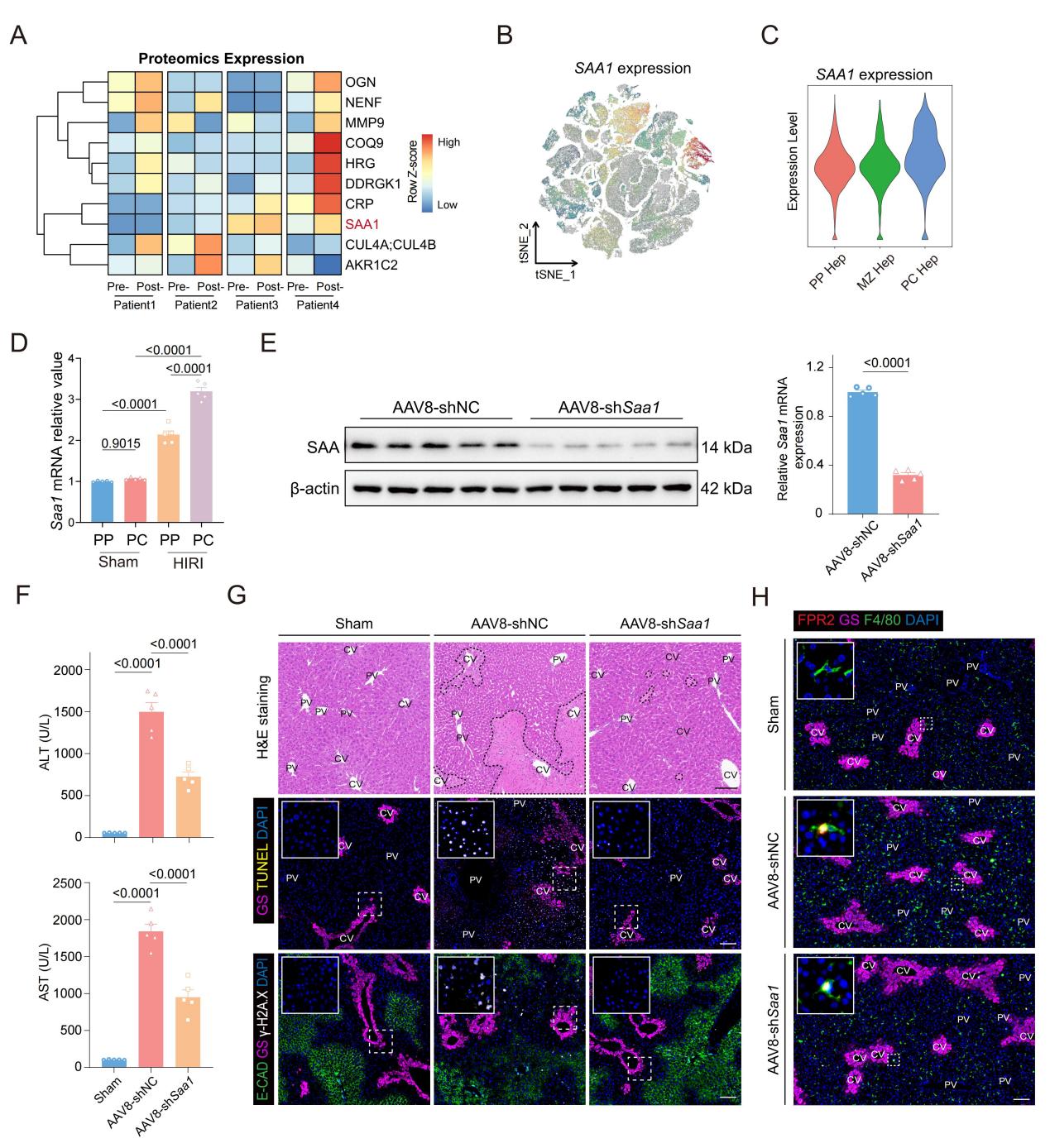


**Supplementary Figure 5. hepatocyte-dominant SAA1 upregulation in post-LT and hepatocyte-specific knockdown**

1. Top 10 proteins upregulated in post-LT donor livers identified by paired differential proteomic analysis of matched normal versus post-LT samples. (B) ScRNA-seq showing that *SAA1* expression is predominantly localized to hepatocytes in human donor livers. (C) ScRNA-seq displaying *SAA1* expression across zonated hepatocyte subsets. (D) RT–qPCR quantification of *Saa1* in PP and PC hepatocytes from sham versus HIRI mouse livers. (E) Immunoblot (left) and RT-qPCR (right) analysis in primary hepatocytes after injection with AAV8-TBG-*Saa1*-shRNA (n = 5, per group). (F) Serum ALT and AST levels (n = 5, per group). (G) H&E (upper), TUNEL (middle), and γ-H2A.X (lower) staining, co-stained with GS and E-CAD in mouse livers. (H) IF staining showing the distribution of FPR2⁺ macrophages around PC hepatocytes. Scale bar, 100 μm. P < 0.05 was considered significant.


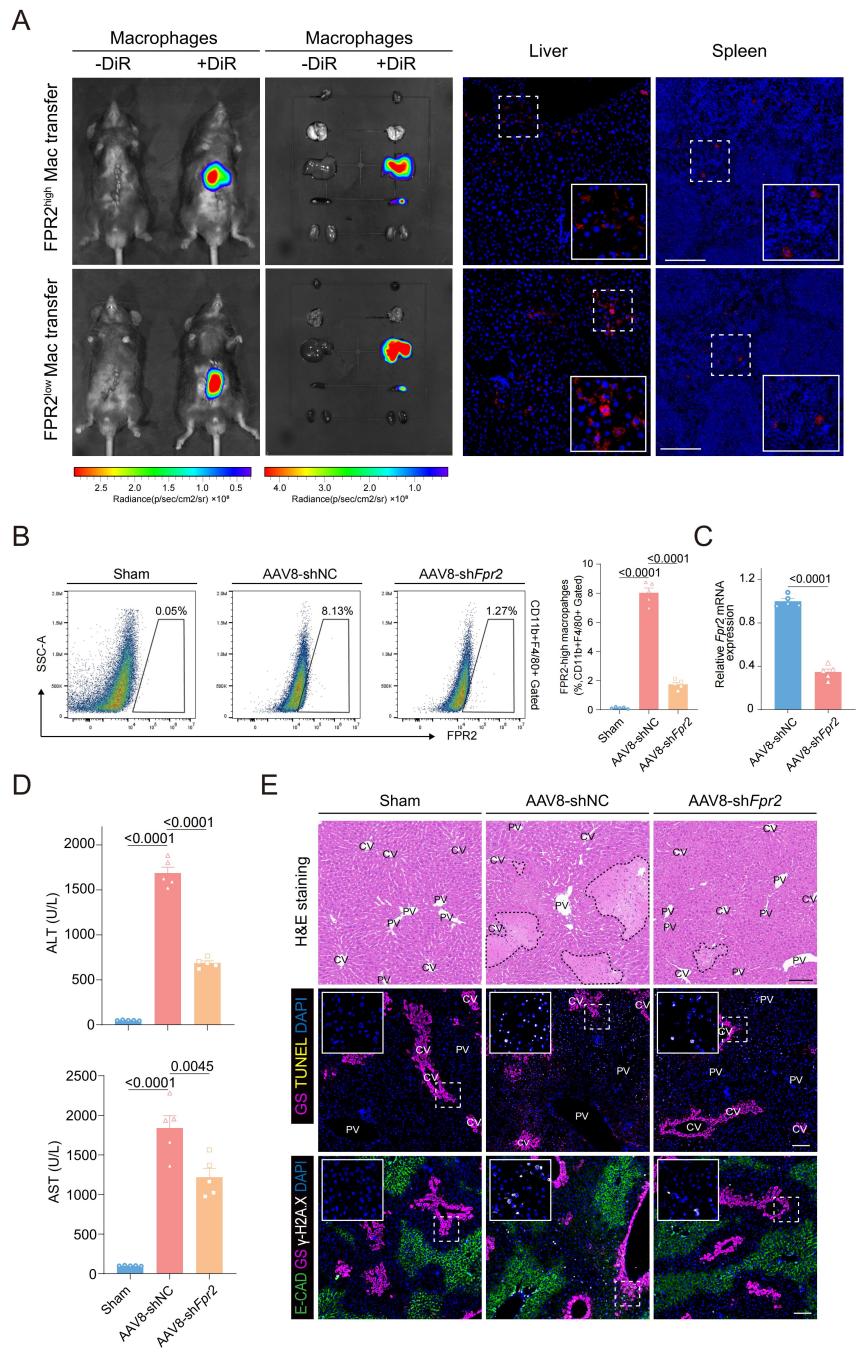


**Supplementary Figure 6. *In vivo* tracking of macrophage transfer and macrophage-specific knockdown of FPR2.**

1. First column: representative IVIS (*In Vivo* Imaging System) images of the mouse HIRI model treated with DiR supernatant (left panel) or DiR-labeled macrophages (right panel) through intravenous injection. Second column: representative IVIS images of five different organs, including the heart, lung, liver, spleen, and kidney, were obtained. Third and fourth columns: Representative fluorescent sections of liver and spleen tissue to detect the distribution of DiR-labeled macrophages (red) were photographed. Scale bar, 50μm. (B) Representative flow-cytometry plots (left) and quantification (right) of FPR2^high^ macrophages after injection with AAV8-F4/80-*Fpr2*-shRNA (n = 5, per group). (C) RT-qPCR analysis of *Fpr2* in primary macrophages isolated from AAV8-F4/80-*Fpr2*-shRNA versus control mice after HIRI (n = 5, per group). (D) Serum ALT and AST levels (n = 5, per group). (E) H&E (upper), TUNEL (middle), and γ-H2A.X (lower) staining, co-stained with GS and E-CAD in mouse livers. Scale bar, 100μm. P < 0.05 was considered significant.


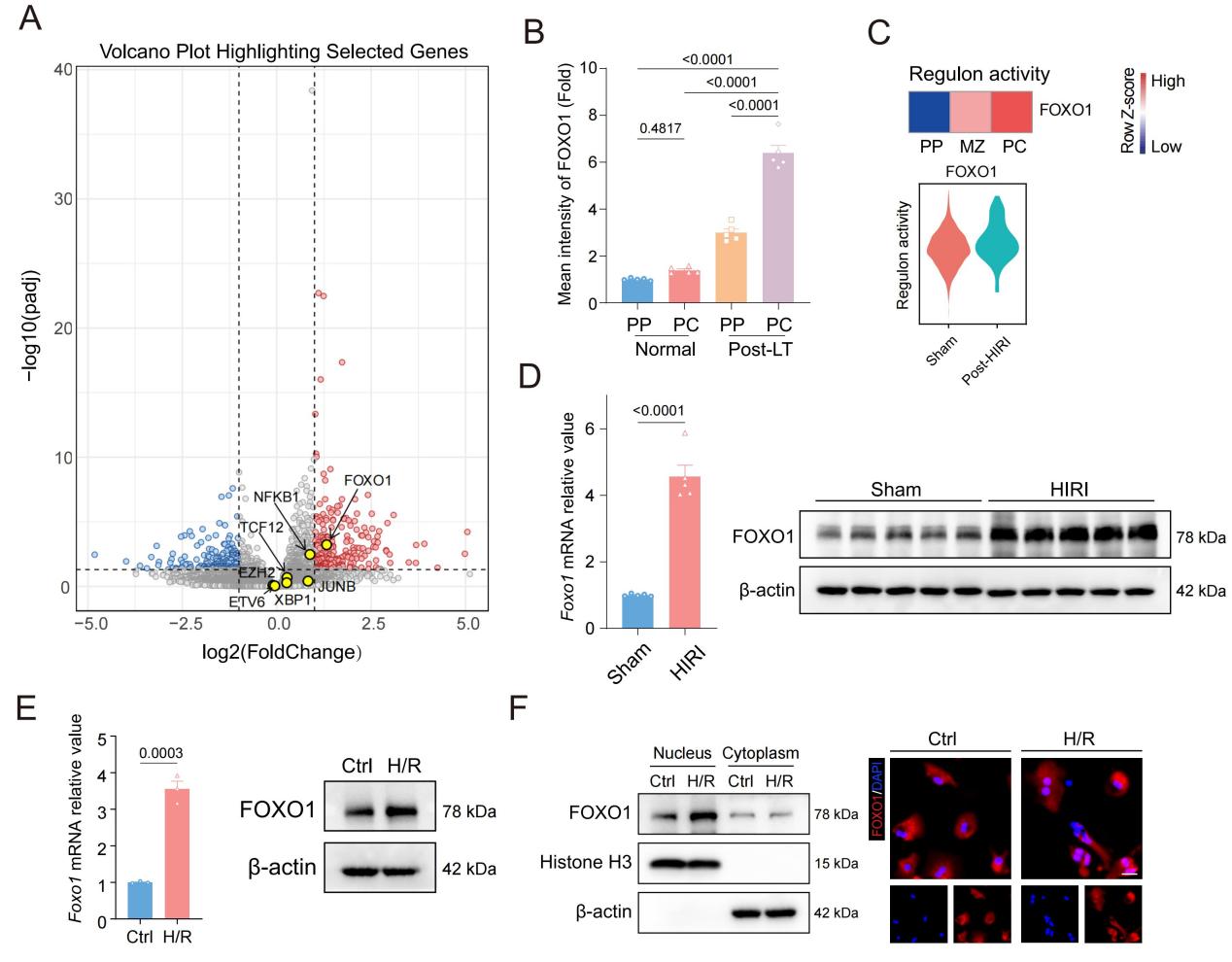


**Supplementary Figure 7. Differential transcription factor expression after liver transplantation and hepatocyte FOXO1 regulon activity with target prediction**

1. Volcano plot showing differential expression of candidate transcription factors in post-LT versus normal human donor livers. (B) Quantification of confocal images showing the zonated distribution of FOXO1 in human liver grafts before and after transplantation (n = 5, per group). (C) FOXO1 regulon activity across zonally defined hepatocytes in the mouse scRNA-seq dataset (upper), and in pericentral hepatocytes from sham and HIRI mouse livers (lower). (D-E) RT-qPCR (left) and Immunoblot (right) analysis of FOXO1 expression in mouse primary hepatocytes isolated from *in vivo* HIRI versus sham models (D, n = 5, per group) and upon *in vitro* hypoxia-reoxygenation (H/R) intervention (E, n = 3, per group). (F) Immunoblot analysis for nuclear/cytoplasmic fractionation (left) and IF staining for subcellular localization (right) of FOXO1 in primary hepatocytes subjected to *in-vitro* H/R intervention. Scale bar, 50 μm. P < 0.05 was considered significant.


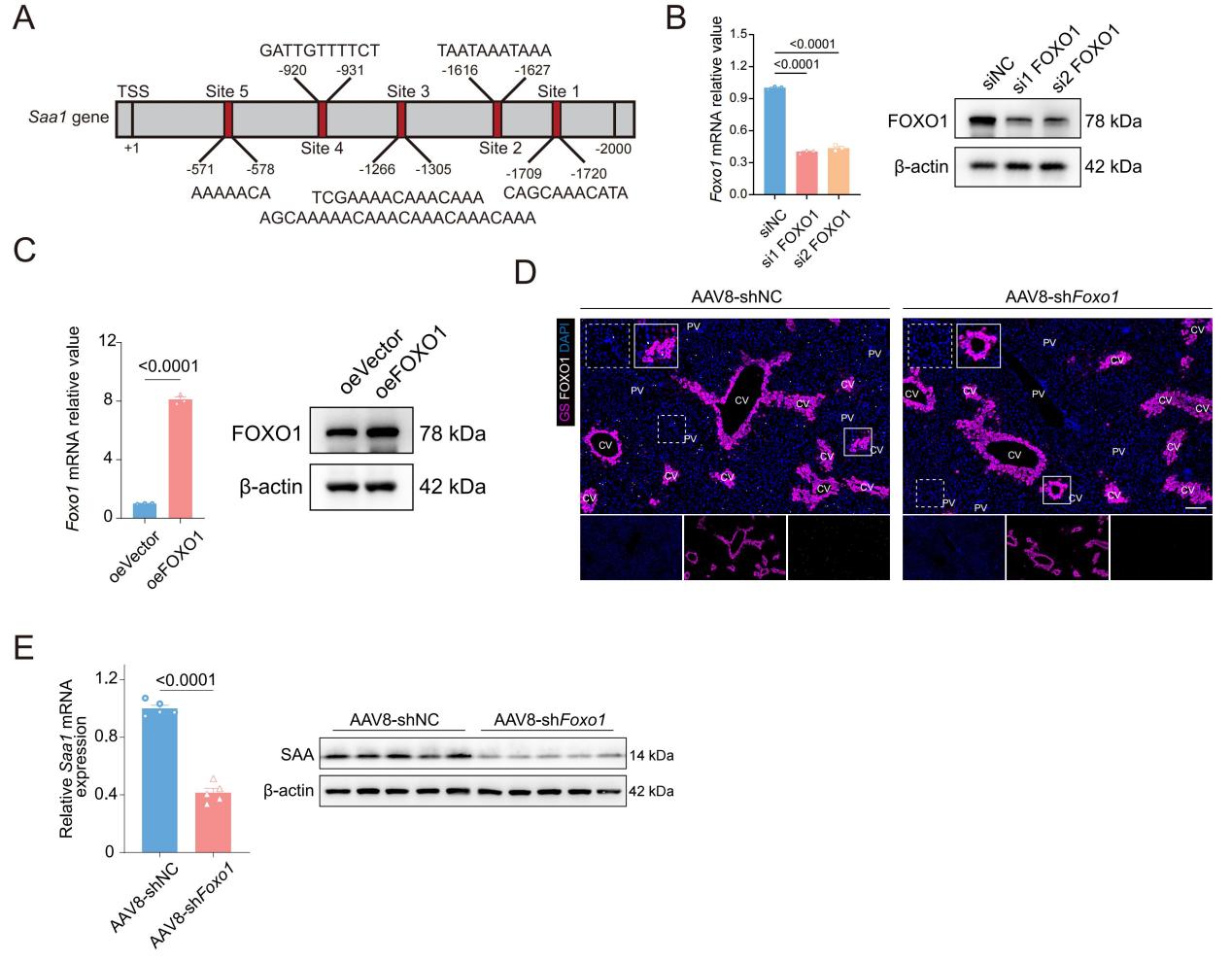


**Supplementary Figure 8. FOXO1 binds the SAA1 promoter and regulates its expression**

1. Predicted FOXO1-binding sites within the mouse *Saa1* promoter identified using the JASPAR database. (B-C) RT-qPCR (left) and Immunoblot (right) analysis of FOXO1 expression in mouse primary hepatocytes upon FOXO1 knockdown (B) or overexpression (C). n = 3, per group. (D) IF staining covered with GS and FOXO1 in mouse livers after injection with AAV8-TBG-*Foxo1-*shRNA. Scale bar, 100 μm. (E) RT-qPCR (left) and Immunoblot (right) analysis of SAA expression in mouse primary hepatocytes after injection with AAV8-TBG-*Foxo1-*shRNA (n = 5, per group). P < 0.05 was considered significant.


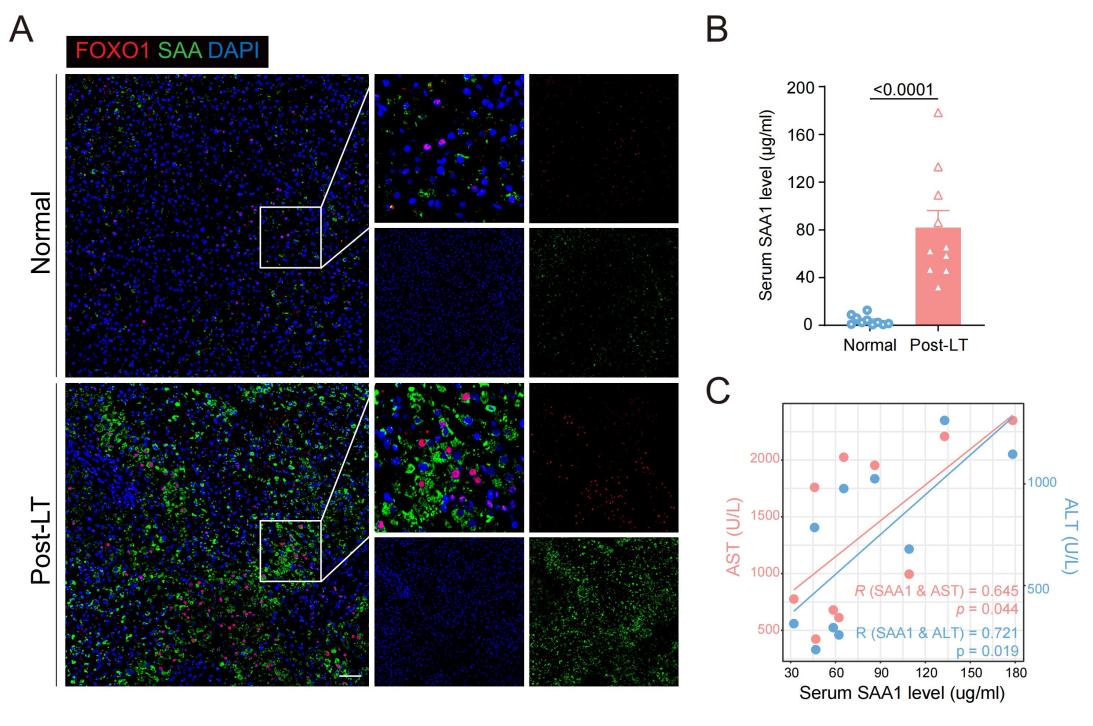


**Supplementary Figure 9. Clinical relevance of SAA to FOXO1 and expression liver function markers in human after LT**

1. IF staining showing FOXO1 and SAA co-localization in human livers after LT. Scale bar, 100 μm. (B) Serum SAA level in human livers before and after LT (n = 10, per group). (C) Correlations between serum SAA and ALT/AST levels in POD1 (n = 10). P < 0.05 was considered significant.


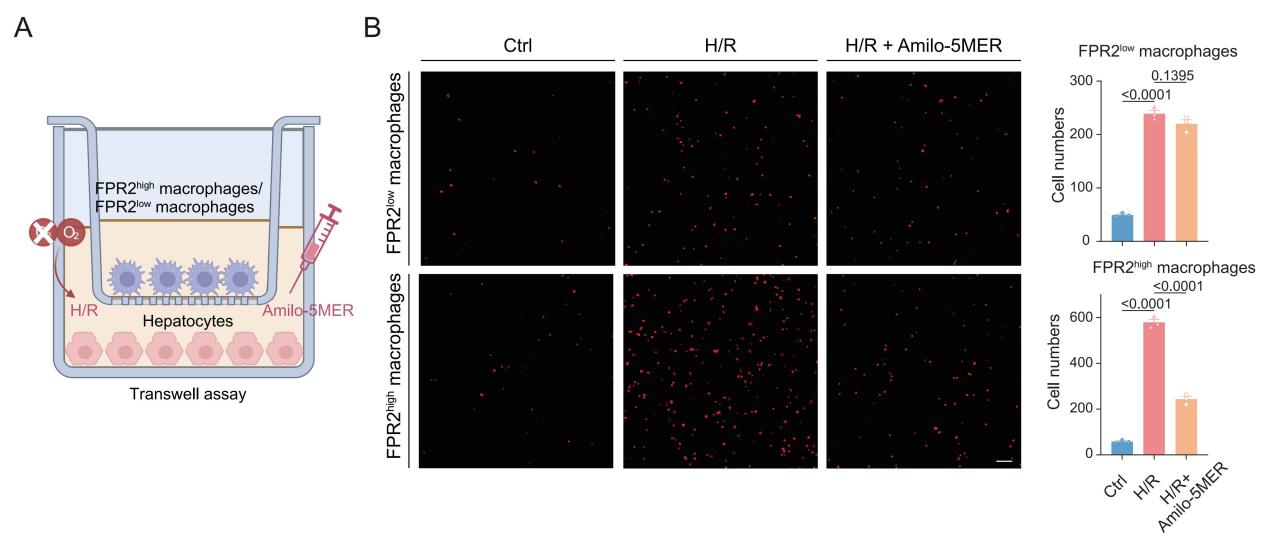


**Supplementary Figure 10. Transwell migration assay to assess the migratory capacity of FPR2^high^ and FPR2^low^ macrophages.**

(A) Schematic displaying the experimental design of the macrophage-migration transwell assay. FPR2^high^ or FPR2^low^ macrophages migrated from the upper to the lower chamber with the stimulation of mouse primary hepatocytes upon H/R and/or Amilo-5MER intervention. (B) Representative images (left) and quantification (right) of F4/80 staining are shown (n = 3, per group). Scale bar, 100μm. P < 0.05 was considered significant.
