# Supplementary material for: SAA/FPR2 Signaling Between Pericentral Hepatocytes and Macrophages Exacerbates Zonated Liver Transplant Injury
Source: Adv Sci (Weinh). 2026 Mar 29;13(34):e22891. doi: 10.1002/advs.202522891 (PMC13285152; doi:10.1002/advs.202522891)
Supplement: Supplementary file 2 — Supporting file: advs75074‐sup‐0001‐TableS1.docx [file ADVS-13-e22891-s001.docx]

**Key resources table.**

- 1. **Antibodies**

| **Name** | **Supplier** | **Cat no.** | **Clonality** |
| --- | --- | --- | --- |
| APC/Cyanine7 anti-mouse CD45 Antibody | BioLegend | 103116 | Monoclonal |
| Brilliant Violet 605™ anti-mouse CD31 Antibody | BioLegend | 102427 | Monoclonal |
| PerCP/Cyanine5.5 anti-mouse/human CD11b Antibody | BioLegend | 101227 | Monoclonal |
| APC anti-mouse F4/80 Antibody | BioLegend | 123115 | Monoclonal |
| FITC anti-mouse FPRL1/FPR2 Antibody | NOVUS | NLS1878F | Monoclonal |
| APC anti-mouse CD73 Antibody | BioLegend | 127210 | Monoclonal |
| PE anti-mouse/human CD324 (E-Cadherin) Antibod | BioLegend | 147304 | Monoclonal |
| TruStain FcX™ PLUS (anti-mouse CD16/32) Antibody | BioLegend | 156603 | Monoclonal |
| Anti-GS | Abcam | ab64613 | Monoclonal |
| Anti-E-cadherin | R&D | AF748 | Polyclonal |
| Anti-γH2A.X | Abcam | ab81299 | Monoclonal |
| Anti-FOXO1 | Abcam | ab39670 | Polyclonal |
| Anti-FOXO1 | CST | 2880 | Monoclonal |
| Anti-F4/80 | Abcam | ab300421 | Monoclonal |
| Anti-CD68 | Affinity | DF7518 | Polyclonal |
| Anti-FPR2 | Abcam | ab203129 | Polyclonal |
| Anti-Histone H3 | CST | 4499 | Monoclonal |
| Anti-β-actin | Immunoway | YM3028 | Monoclonal |
| Anti-SAA | Proteintech | 13192-1-AP | Polyclonal |
| Anti-SAA | R&D | AF2948 | Polyclonal |
| HRP-conjugated goat anti-rabbit IgG (H+L) | CST | 7074 | Polyclonal |
| HRP-conjugated horse anti-mouse IgG (H+L) | CST | 7076 | Polyclonal |
| Donkey anti-Mouse IgG (H+L), Alexa Fluor™ 488 | invitrogen | A21202 | Polyclonal |
| Goat anti-Rabbit IgG (H+L), Alexa Fluor™ 594 | invitrogen | A11012 | Polyclonal |
| Goat anti-Mouse IgG (H+L), Alexa Fluor™ 647 | invitrogen | A21235 | Polyclonal |

- 1. **Organisms**

| **Name** | **Supplier** | **Strain** | **Sex** | **Age** |
| --- | --- | --- | --- | --- |
| C57BL/6 mice | Jiangsu Wukong Biotechnology | C57BL/6 | Male | 8 weeks |

- 1. **Sequence-based reagents**

| **Name** | **Sequence** | **Supplier** |
| --- | --- | --- |
| m*Tnf*_qF | CCCTCACACTCAGATCATCTTCT | BGI Genomics |
| m*Tnf*_qR | GCTACGACGTGGGCTACAG | BGI Genomics |
| m*Il1b*_qF | GCAACTGTTCCTGAACTCAACT | BGI Genomics |
| m*Il1b*_qR | ATCTTTTGGGGTCCGTCAACT | BGI Genomics |
| m*Il6*_qF | TAGTCCTTCCTACCCCAATTTCC | BGI Genomics |
| m*Il6*_qR | TTGGTCCTTAGCCACTCCTTC | BGI Genomics |
| m*Saa1*_qF | AGACACCAGCAGGATGAAGC | BGI Genomics |
| m*Saa1*_qR | TCATGTCAGTGTAGGCTCGC | BGI Genomics |
| m*Fpr2*_qF | GAGCCTGGCTAGGAAGGTG | BGI Genomics |
| m*Fpr2*_qR | TGCTGAAACCAATAAGGAACCTG | BGI Genomics |
| m*Foxo1*_qF | CCCAGGCCGGAGTTTAACC | BGI Genomics |
| m*Foxo1*_qR | GTTGCTCATAAAGTCGGTGCT | BGI Genomics |
| m*Cyp2e1*_qF | CGTTGCCTTGCTTGTCTGGA | BGI Genomics |
| m*Cyp2e1*_qR | AAGAAAGGAATTGGGAAAGGTCC | BGI Genomics |
| m*Sds*_qF | GAAGACCCCACTTCGTGACAG | BGI Genomics |
| m*Sds*_qR | TCTTGCAGAGATGCCCAATGC | BGI Genomics |
| m*β-actin*_qF | ACTGTCGAGTCGCGTCCA | BGI Genomics |
| m*β-actin*_qR | TCATCCATGGCGAACTGGTG | BGI Genomics |
| m*Saa1*_Site1_qF | GATGGCTCAGCAGGTCAG | BGI Genomics |
| m*Saa1*_Site1_qR | ATCTCATTACGGATGGTTGTG | BGI Genomics |
| m*Saa1*_Site2_qF | GCTACAGTGTACTTAGATA | BGI Genomics |
| m*Saa1*_Site2_qR | GTTGTTGGGAATTGAATT | BGI Genomics |
| m*Saa1*_Site3_qF | AAGTAAGTTCCAGGACAG | BGI Genomics |
| m*Saa1*_Site3_qR | GGTATGAATGAGAAGAAGAC | BGI Genomics |
| m*Saa1*_Site4_qF | GGTTAAGAAGCACAGGAC | BGI Genomics |
| m*Saa1*_Site4_qR | TGTCACACCAGTCTCTATG | BGI Genomics |
| m*Saa1*_Site5_qF | TGCTAGTAGAAGCCTCTT | BGI Genomics |
| m*Saa1*_Site5_qR | CACCATTCTCTGACTGATAT | BGI Genomics |

| **siRNA** | **sense** | **antisense** |
| --- | --- | --- |
| *Foxo1*- Mus-si1 | GCAACGAUGACUUUGAUAATT | UUAUCAAAGUCAUCGUUGCTT |
| *Foxo1*- Mus-si2 | GCCAAACACCAGUCUAAAUTT | AUUUAGACUGGUGUUUGGCTT |
| *Saa1*- Mus-si | CCUGACAAAUACUGAGCGUTT | ACGCUCAGUAUUUCUCAGGTT |
| *Fpr2*- Mus-si | GGUCAAACCAGUGAUUCAATT | UUGAAUCACUGGUUUGACCTT |

- 1. **Other (e.g. drugs, proteins, vectors etc.)**

| **Name** | **Supplier** | **Cat no.** |
| --- | --- | --- |
| HBSS Ca^2+^(-), Mg^2+^(-) | Gibco | C14175500BT |
| HBSS Ca^2+^(+), Mg^2+^(+) | Gibco | 14025092 |
| Collagenase IV | Sigma‒Aldrich | C4-28-100MG |
| DMEM/F-12 | Gibco | C11330500BT |
| RPMI-1640 | Gibco | C11875500BT |
| DMEM | Gibco | C11995500BT |
| FBS | Gibco | 10099141C |
| EGTA solution | Beyotime | ST068 |
| Percoll | Biosharp | BS909 |
| penicillin-streptomycin | Gibco | 15140122 |
| ITS-G | BasalMedia | S450J7 |
| JetPRIME | Polyplus | 101000046 |
| Mouse SAA1 ELISA Kit | MEIMIAN | MM-46455M2 |
| Human SAA1 ELISA kit | Biodragon | BDEL0724-96T |
| Dako REAL™ EnVision™ Detection System | Dako | K5007 |
| Evo M-MLV RT Mix Kit with gDNA Clean for qPCR | AG | AG11728 |
| SYBR qPCR SuperMix Plus | Novoprotein | E096-01B |
| NE-PER Nuclear & Cytoplasmic Extraction Kit | Thermo Fisher | 78835 |
| ChIP Assay Kit | Beyotime | P2078 |
| Amilo-5MER | MedChemExpress | HY-P10935A |
| DMSO | Sigma-Aldrich | D5879 |
| SBE-β-CD | MedChemExpress | HY-17031 |
| One-step TUNEL apoptosis detection kit | Beyotime | C1088 |
| Antifade mounting medium with DAPI | KeyGEN | KGF0282 |
| Zombie Aqua™ Fixable Viability Kit | BioLegend | 423101 |
| Zombie Green™ Fixable Viability Kit | BioLegend | 423111 |
| Clodronate Liposomes | FormuMax Scientific | F70101C-N |
| DiR | Thermo Fisher | D12731 |
